# Biocomplexity in Populations of European Anchovy in the Adriatic Sea

**DOI:** 10.1371/journal.pone.0153061

**Published:** 2016-04-13

**Authors:** Paolo Ruggeri, Andrea Splendiani, Giulia Occhipinti, Tatiana Fioravanti, Alberto Santojanni, Iole Leonori, Andrea De Felice, Enrico Arneri, Gabriele Procaccini, Gaetano Catanese, Vjekoslav Tičina, Angelo Bonanno, Paola Nisi Cerioni, Massimo Giovannotti, William Stewart Grant, Vincenzo Caputo Barucchi

**Affiliations:** 1 Dipartimento di Scienze della Vita e dell’Ambiente, Università Politecnica delle Marche, Via Brecce Bianche, 60131, Ancona, Italy; 2 Consiglio Nazionale delle Ricerche, Istituto di Scienze Marine Sezione Pesca Marittima, Largo Fiera della Pesca, 60125, Ancona, Italy; 3 FAO-FIRF, Fisheries and Aquaculture Department, AdriaMed Project, Viale delle Terme di Caracalla, 00153, Roma, Italy; 4 Stazione Zoologica Anton Dohrn, Villa Comunale, 80121, Napoli, Italy; 5 Institute of Oceanography and Fisheries, Laboratory of Fisheries Science and Management of Pelagic and Demersal Resources, Šetalište I. Meštrovića, 63, 21000, Split, Croatia; 6 Istituto per l’Ambiente Marino Costiero (IAMC), Consiglio Nazionale delle Ricerche (CNR), Detached Unit of Capo Granitola (TP), Via del Mare, 3 91021, Torretta Granitola - Fraz. di Campobello di Mazara (TP), Italy; 7 Commercial Fishery Division, Alaska Department of Fish and Game, Commercial Fisheries Division, Anchorage, 99518, AK, United States of America; University of Padova, ITALY

## Abstract

The sustained exploitation of marine populations requires an understanding of a species' adaptive seascape so that populations can track environmental changes from short- and long-term climate cycles and from human development. The analysis of the distributions of genetic markers among populations, together with correlates of life-history and environmental variability, can provide insights into the extent of adaptive variation. Here, we examined genetic variability among populations of mature European anchovies (*n* = 531) in the Adriatic (13 samples) and Tyrrhenian seas (2 samples) with neutral and putative non-neutral microsatellite loci. These genetic markers failed to confirm the occurrence of two anchovy species in the Adriatic Sea, as previously postulated. However, we found fine-scale population structure in the Adriatic, especially in northern areas, that was associated with four of the 13 environmental variables tested. Geographic gradients in sea temperature, salinity and dissolved oxygen appear to drive adaptive differences in spawning time and early larval development among populations. Resolving adaptive seascapes in Adriatic anchovies provides a means to understand mechanisms underpinning local adaptation and a basis for optimizing exploitation strategies for sustainable harvests.

## Introduction

A growing body of evidence indicates that marine organisms with potentially dispersive planktonic larvae can show genetically patchy distributions that may be uncorrelated with spatial complexity [1, 2]. This unexpected genetic population structure has been attributed to natural selection on life-history stages [3, 4], on isolation by hydrographic barriers to gene flow [5] and on adaptation to local environments [6]. The interplay between these forces is referred to as biocomplexity, which underpins population resilience and persistence [7–9]. The use of ocean-wide environmental databases and genetic markers to understand the origins of biocomplexity is a relatively new area known as seascape genetics [10, 11]. Spatial patterns among marine populations can provide insights into interactions between environmental variables and life-history traits and can illuminate the roles of these variables in shaping genetic structure. Marine ecosystems are dynamic and complex, reflecting interactions between biotic and abiotic variables [11]. Small-scale spatial and temporal heterogeneity can impose selective constraints on the development and survival of larvae and juveniles [11, 12] and hence on the distributions of adults [13–15].

Here, we focus on anchovy (*Engraulis encrasicolus* L.) populations in the Adriatic Sea. This species support large fisheries, as well as artisanal harvests along the coasts of Italy and the Balkan countries. The Adriatic is a semi-enclosed basin open to the Central-Eastern Mediterranean Sea and is divided into several areas with contrasting levels of productivity [16]. Four areas with different local hydrographic and physical properties can be recognized [17, 18]. The first area (Zone A, *sensu* [16]) encompasses deep, oligotrophic waters in the southern central Adriatic and receives warm, high saline water from the Eastern Mediterranean Sea. A second area (Zone B) lies in shallow waters (mean depth < 40–50 m) of the northwestern Adriatic and receives large amounts of freshwater from the western side of the Adriatic. Zone B is characterized by high levels of productivity and strong seasonal swings in temperature, salinity and dissolved oxygen. A third area (Zone C) lies in the northeastern Adriatic Sea with depths greater than those in the western Adriatic, moderate levels of productivity, high salinities and stable environmental conditions throughout the year. A fourth area (Zone D) includes the lagoons and channels on both sides of the Adriatic. These areas have the highest levels of productivity in the Adriatic Sea, but represent only 1–2% of its surface [16–18].

High levels of productivity in the northern Adriatic basin support spawning and feeding grounds for many marine species of economic and ecological importance. One of these is the European anchovy which spawns several times from April to October in defined spawning areas [19]. Potential spawning and juvenile nursery areas are located over the continental shelf in the northwestern Adriatic, where high inshore productivity enhances larval growth and promotes the recruitment of juveniles into adult populations [20].

Two anchovy stocks in the Adriatic were previously described based on color [21] and allozyme-frequency variation [22]. A small silver anchovy occurs largely in the shallow and less saline waters of the northern Adriatic, and a larger bluish form inhabits the open waters of the central basin, for the most part. These morphs are superficially similar to *E*. *albidus* (silver anchovy) and *E*. *encrasicolus* (blue anchovy) in the Gulf of Lyon, and led Borsa *et al*. [21] to suggest that both species also inhabit the Adriatic Sea. Additionally, Bembo *et al*. [22] found strong allozyme-frequency gradients among populations in the northwestern and southeastern areas of the Adriatic. However, the distributions of these gradients did not correspond entirely with the distributions of the two putative species. In the interim, no studies have been made with additional molecular markers to investigate these differences.

Here, we used microsatellite DNA markers to resolve the fine-scale genetic structure of anchovy populations in the Adriatic. Among the molecular markers used for population genetic studies, microsatellite DNA has proved to be a powerful marker in studies of population structure at regional and sub-basin scales due to its high level of polymorphism (reviewed in [23]). We had four objectives: i) test whether multiple species of anchovies exist in the Adriatic basin as postulated by Borsa *et al*. [21], ii) identify genetically discrete populations and local stocks, iii) estimate the directionality of gene flow and the level of connectivity among populations and iv) identify environmental variables influencing the adaptive seascape of Adriatic anchovies.

## Materials and Methods

### Ethical statement, Sample collection and DNA extraction

**Fig 1:**
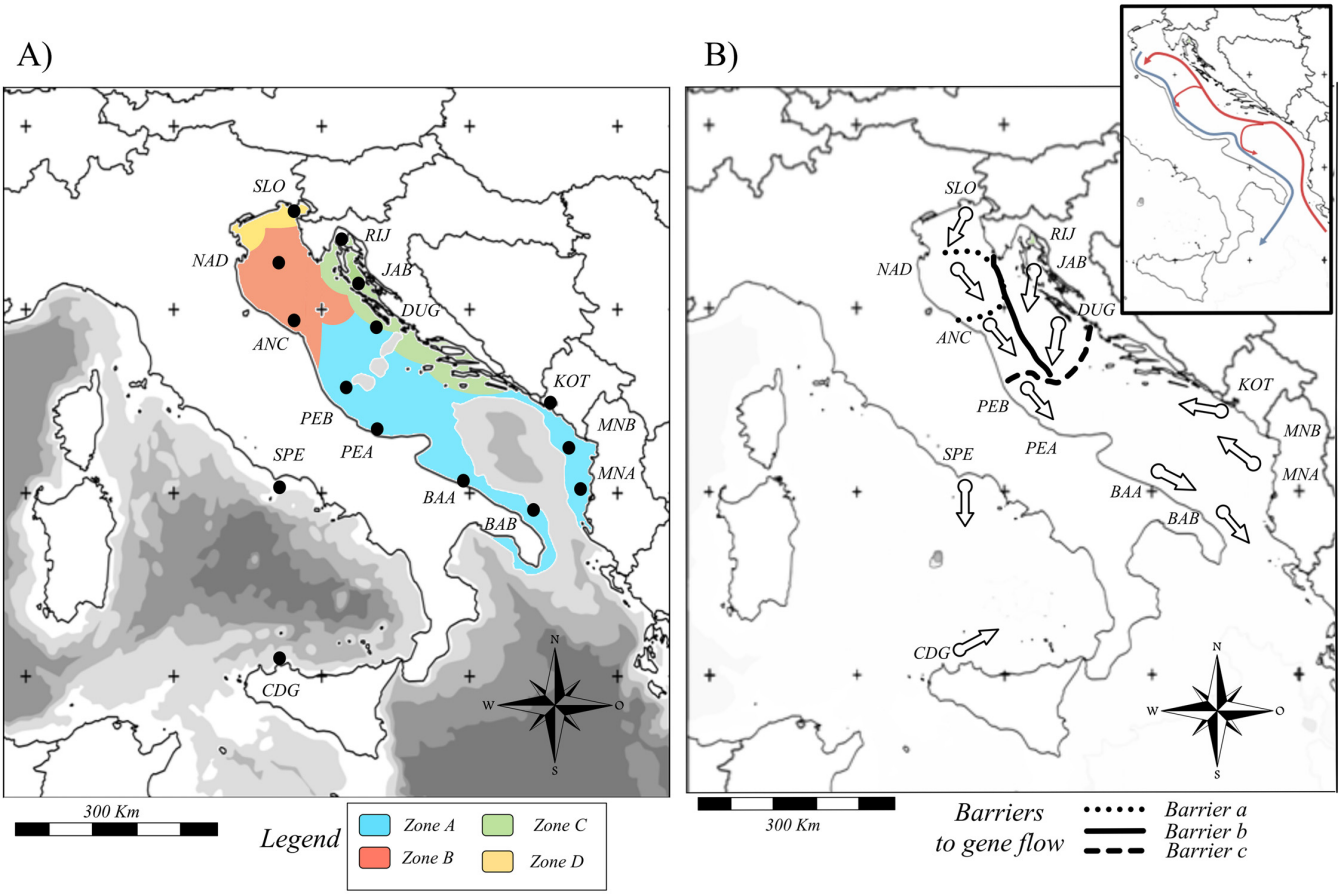
Maps showing sample locations. (a) Map of the Adriatic and Tyrrhenian Seas showing the sampling localities and a general representation of the four main areas described by [17, 18]. (b) Map showing outcomes from the BARRIER [50] and Migrate-n [54]. The three lines (solid, dotted and dashed) represent major barriers to dispersal. The arrows represent the directionality of gene flow from each locality and a map (in the right up corner) of major sea-surface currents allows a direct comparison among them.

A total of 531 mature European anchovies were collected at 13 localities in the Adriatic between July and September 2012 and at two localities in the Tyrrhenian Sea between May and June 2013 (Fig 1A; Table 1A). Since samples of European anchovies were collected during research cruises, special ethical permits for scientific sampling were not required. Samples from the Adriatic, except the Boka Kotorska sample (KOT), were collected by midwater pelagic trawls during annual acoustic surveys between July and September 2012 in southern (July) and northern-central (September) areas of the Adriatic Sea. Tyrrhenian samples were collected with pelagic trawls during the EVATIR 2013 oceanographic research cruise that took place May–June 2013. No area under protection or special management was included in our sampling design. Permission to carry out surveys in Italian waters was granted by the MIPAAF “Ministero per le Politiche Alimentari, Agricole e Forestali”, Stato Maggiore della Marina (MARISTAT), Istituto Maridrografico, Comandi Militari Marittimi di La Spezia and Taranto and the local Adriatic/Tyrrhenian Port Authorities. Sampling in the waters of Montenegro was permitted by the Local Ministry of Agriculture and Rural Development, and sampling off Albania was permitted by the Ministria Mjedisit, Pyjeve dhe Administrimit Ujrave Drejtorise Politikave te Peshkimit. Permission to conduct the acoustic survey and related biological samplings of small pelagic fish in the eastern part of the Adriatic Sea during September 2012 on board the r/v BIOS DVA was granted by the Croatian Ministry of Agriculture and Ministry of science, education and sports. Whole animals were preserved in absolute or 95% ethanol at -20°C until DNA extraction. Total DNA was extracted from gill or muscle tissue using a standard phenol-chloroform protocol (gill) [24], or with the NucleoSpin Tissue Kit (muscle) (Macherey-Nagel).

**Table 1:** Sample locations, dates, sample sizes and summary statistics by population. Basin, A = Adriatic Sea; T = Tyrrhenian Sea; N = Number of individuals sampled; Mean *N*_A_ = mean number of alleles; *R*_S_ = allelic richness; *H*_E_ = Expected heterozygosity by population; *H*_O_ = Observed heterozygosity.

Basin	Locality	Sample label	N	Coordinates	Sampling depth (m)	Bottom depth (m)	Sampling date (month/year)	Mean *N*_A_	*R*_S_	*H*_E_	*H*_O_
A	Slovenja-Piran	SLO	35	45°33.57’N; 13°35.64’E	12	22.5	Sep-12	14.2	9.8	0.826	0.791
A	Croatia- Rijeka	RIJ	35	45°16,53'N; 14°25,07'E	40	64	Sep-12	11.5	9.6	0.763	0.728
A	Northern Adriatic Sea	NAD	35	44°49.97'N; 13°13.26'E	30	41	Sep-12	12.2	8.6	0.722	0.751
A	Croatia- Jablanac	JAB	35	44°41,47'N; 14°53,32'E	83	104	Sep-12	11.4	8.2	0.749	0.742
A	Croatia- Dugi Otok	DUG	35	43°53,07'N; 14°55,66'E	61	75	Sep-12	12.2	8.1	0.763	0.776
A	Ancona	ANC	35	43°42.20’N; 13°37.76’E	34	38.4	Sep-12	13.5	8.4	0.818	0.752
A	Pescara	PEB	35	42°54.19’N; 14°10.31’E	58	70.8	Sep-12	13.9	9.3	0.818	0.78
A	Pescara	PEA	35	42°30.19’N; 14°18.14’E	8	24.1	Sep-12	13.9	9.8	0.811	0.781
A	Boka Kotorska	KOT	50	42°25.55’N; 18°39.48’E	20	40	Jul-12	15.8	9.8	0.808	0.799
A	Montenegro	MNB	37	41°53.98’N; 19°00.96’E	75	86.7	Aug-12	13.9	9.7	0.821	0.756
A	Montenegro	MNA	35	41°49.69’N; 19°16.06’E	16	52.4	Aug-12	14	9.8	0.815	0.753
A	Bari	BAA	35	41°15.32’N; 16°34.13’E	23	31.6	Jul-12	12.8	9.2	0.809	0.758
T	Sperlonga	SPE	30	41°12.17’N; 13°23.85’E	40	80	Jun-13	12.5	10.7	0.792	0.75
A	Bari	BAB	35	41°10.60’N; 17°07.80’E	115	125.3	Jul-12	13.2	9.4	0.811	0.734
T	Castellammare del Golfo	CDG	29	38°04.81’N; 12°59.29’E	20	40	May-13	13.2	11.2	0.792	0.753

### Molecular markers and PCR amplification

All samples were screened at 14 previously described microsatellite loci [25–28]. Eleven loci were labeled with fluorescent dyes and multiplexed in three polymerase chain reactions (PCR) (S1 Table, S1 File). These loci were genotyped using an ABI-PRISM 3130xl Genetic Analyzer (Applied Biosystems). The remaining loci, Enja-148, Ee2-508 and Ee2-165b, were amplified individually due to moderate levels of polymorphism and were screened with a silver staining protocol [29]. Additional information about PCR protocols, amplification profiles, fragment genotyping, internal standards, silver staining adopted for the non-labeled PCR products and genotyping accuracy are provided in S1 File.

### Statistical treatment of microsatellite and mtDNA data

The incidences of null alleles, allelic dropout and stutter peaks were assessed with MICROCHECKER 2.2.1 [30], and loci affected by null alleles were corrected with the Brookfield algorithm [31] in MICROCHECKER. In addition, the Dempster algorithm [32] in FreeNa [33] was used to estimate null allele frequencies per locus and per sampling locality. Number of alleles per locus (*N*_A_), allelic richness (*R*_S_), observed (*H*_O_) and expected (*H*_E_) heterozygosity, and the inbreeding coefficient (*F*_IS_) were calculated for each sample using FSTAT 2.9.3 [34].

Conformation to Hardy-Weinberg proportions was tested with the exact test implemented in Genepop 4.0.10 [35] using a Markov Chain Monte Carlo (MCMC) method with 100 batches of 10 000 iterations each, with the first 1000 iterations discarded before sampling. Genepop was also used to test for linkage disequilibrium between loci by a MCMC chain executed with 1000 batches of 2000 iterations each. A sequential Bonferroni adjustment of *P*-values was used to account for an increase in type-I error for multiple comparisons [36].

Genetic differentiation between samples was estimated with Ɵ_ST_ [37] and compared with divergences (*F*_ST_) estimated with the Exclusion of Null Allele Method (ENA) in FreeNa [33]. High heterozygosity and large number of alleles (20 or more) can inflate estimates of *F*_ST_ [38]. To evaluate this effect, genetic differentiation between samples was also estimated with *G*_ST_ [39], *G*'_ST_ [40] and *D*_EST_ [41]. *G*_ST_, *G*'_ST_ and *D*_EST_ were calculated with GenAlEx 6.5 [42]. Genetic population structure was also examined using a Bayesian approach implemented in STRUCTURE 2.3.2.1 [43, 44] and by the multivariate ordination method in DAPC [45], implemented in the adegenet package [46] in R 2.15.3 (R Development Core Team, 2009; http://www.r-project.org).

Runs in STRUCTURE were made assuming *K* = 1–10 and imposing an admixture model with correlated allele frequencies. Each *K* value was replicated with ten independent runs of 10^6^ MCMC iterations, after a burn-in of 10^5^ iterations. A set of STRUCTURE simulations were made using i) all loci, ii) only neutral loci and iii) only candidate outlier loci. These simulations were repeated with (LocPrior mode) and without Prior information. When Prior information was included, the following clustering criterion (based on geographical partition of data) was assumed: Cluster 1 (northwest Adriatic) [SLO+NAD+ANC+PEA+PEB]; Cluster 2 (southwest Adriatic) [BAA+BAB]; Cluster 3 (southeast Adriatic) [KOT+MNA+MNB]; Cluster 4 (northeast Adriatic) [RIJ+JAB+DUG] and Cluster 5 (Tyrrhenian Sea) [SPE+CDG]. The Evanno method [47] in STRUCTURE HARVESTER [48] was used to determine the most likely *K* value from all simulations and graphical representation of multiple runs per *K* values were produced by the CLUMPAK Server (http://clumpak.tau.ac.il) [49].

DAPC [45] is a multivariate approach using principal components of genetic variation (PCA) that maximizes differences among groups while minimizing differences within groups. A set of 150 principal components was retained as predictors for discriminant analysis among samples.

The geographic boundaries among sampled populations were identified with BARRIER 2.2 [50], which uses Monmonier’s algorithm to identify boundaries characterized by an abrupt break in gene flow (genetic barriers) and plots these boundaries on a map with sample coordinates. The strengths of putative boundaries were evaluated using a bootstrapping procedure separately for each locus.

Contemporary gene flow was evaluated with an individual-assignment test in GENECLASS 2.0 [51]. For each locality, the criterion of Rannala and Mountain [52] was applied using 10 000 individuals and the Paetkau *et al*. [53] algorithm to detect first generation migrants.

The second method used to detect gene flow and connectivity was based on the coalescent calculation of historical migration rates between sampling locations using MIGRATE-n 3.5.1 (http://popgen.sc.fsu.edu/Migrate/Migrate-n.html). A Bayesian method was applied [54] and *F*_ST_ estimates among localities were used as baseline to calculate the mutation parameter Θ (Θ = 4*N*_e_μ, where *N*_e_ is the effective population size and μ is the locus mutation rate) and the historical migration rate, *M* (*M* = *m*/μ, where *m* is the immigration rate per generation). A Brownian motion model was used and mutation rates were assumed to be constant over a set of 1000 neutral loci. The MCMC procedure consisted of one long chain of 500 000 genealogies for each locus, with the firs 10 000 genealogies discarded as burn-in. The sum of *M* values from each location was used to determine directionality of gene flow.

The relationship between genetic and geographic distances between samples (isolation by distance) was tested using Mantel's difference matrix test implemented in Genepop (ISOLDE) [35]. Genetic distances (Ɵ_ST_) between samples were compared to linear shortest sea-distances between samples with a permutation test of 10 000 iterations.

Non-neutral loci were identified with four methods that are based on different statistical assumptions. First, the coalescent approach in LOSITAN [55] detects outlier loci from the joint distribution of *F*_ST_ and expected heterozygosity (*H*_E_) under the island model of migration [56]. Runs were made with both the Infinite Allele Mutation Model (IAM) and Stepwise Mutation Model (SMM) each with 100 000 simulations. A second, Bayesian approach implemented in BayeScan 2.0 [57], uses a Reversible Jump Markov Chain Monte Carlo (RJ-MCMC) algorithm to obtain posterior distributions and is useful for small sample sizes because it reduces bias. We used default parameters of 20 independent runs with 5000 iterations each, 50 000 iterations burn-in, sample sizes of 5 000 and thinning of 10. BayeScan results were inferred following the Jeffrey’s interpretation, as described in the BayeScan User Manual. Third, a hierarchical finite island model [58] in Arlequin 3.5.1.3 [59] was used to identify potential candidate loci by partitioning the dataset into population clusters as inferred from the STRUCTURE results. This method is particularly efficient for species showing significant population structure, which reduces false positives in the hierarchical analysis of genetic differentiation. Replicate coalescent runs of 100 demes were performed with 20 000 iterations. Only loci identified as outliers in all three approaches were considered as true outliers. Forth, we used the LnHR method [60], which is specifically designed to detect outlier microsatellites by evaluating changes in *H*_E_ between pair of samples under the following formula:

$$LnRHpopA-popB=Ln\left[{\left(\frac{1}{1-HeA}\right)}^{2}-{\left(\frac{1}{1-HeB}\right)}^{2}-1\right]$$

We used a threshold of ±2.58 and assumed that LnRH values under neutrality were normally distributed after data standardization (mean = 0, SD = 1). The values of 99% of neutral loci are expected to fall within this range.

A set of 13 environmental variables (listed in S2 Table) was recovered from the Sea Data-Net Climatologies Pan-European Infrastructure for Ocean and Marine Data Management web site (http://gher-diva.phys.ulg.ac.be/web-vis/). In this database, chlorophyll and oxygen values were reported as averages over 1890 and 2008, whereas salinities and temperatures were reported as averages between 1900 and 2009. These values were used as independent variables in a redundancy discriminant analysis (RDA) implemented in CANOCO 4.5 [61]. These ordinations were compared to the allelic-frequencies distributions of outlier loci to test for allele-environment associations. Alleles with frequencies less than 5% were removed to reduce noise, and the environmental data series was tested for a neutral distribution by a Shapiro-Wilk test. Non-neutral variables were Log_10_ or Ln transformed for seascape analyses. The forward selection of environmental variables at each locus was performed with a Monte Carlo permutation test of 999 iterations (*P* < 0.01). When a locus was associated with more than one variable, a permutation test as above was repeated to establish the conjunct significance of these variables on the distribution of allele frequencies. The associations between allele frequencies in samples and environmental variables were further investigated with a Canonical Correspondence Analysis (CCA) in PAST 2.17 [62] with 10 000 permutations to determine significance.

### Detection of multiple anchovy species in the Adriatic Sea

Based on the assumptions of Borsa *et al*. [21], the most important feature distinguishing shallow-water silver anchovies (*E*. *albidus* sensu Borsa *et al*. [21]) from open-water blue anchovies (*E*. *encrasicolus*) is bathymetry. Hence, we collected samples from shallow coastal sites (<50m) and from open-water areas (>50m). We first performed a STRUCTURE simulation to test the effect of bathymetry. Two major groups of samples were defined based on the threshold of 50 m of the bottom depth and were used as *a priori* information. Cluster 1 included SLO+NAD+ANC+PEA+KOT +BAA+CDG, and Cluster 2 included the remaining samples. Second, we verified the consistency of clustering samples based on their sampling bathymetry (as performed in STRUCTURE) using an AMOVA test. Finally, estimates of genetic differentiation between samples were used to test for large genetic differences, as expected between divergent species.

## Results

### Microsatellite variability

The PCR success rate was high for the 14 microsatellite loci with only 1.17% missing genotypes. Although allele dropout and stuttering were minimal, null alleles were detected in 50 of 210 global tests. Null-allele corrections with the Brookfield algorithm improved the results, but 26 of 210 tests were still significant. Ten of the significant tests were for Enja148, which we excluded from the following analyses.

Locus polymorphism varied from 11 alleles at Ee2-508 to 41 alleles at Ee2-507 (S3 Table). The mean number of alleles (*N*_A_) per sample over all loci varied from 11.4 alleles in JAB to 15.8 alleles in KOT. Allelic richness (*R*_S_), standardized with 19 individuals per sample, varied from 8.1 (DUG) to 11.2 (CDG) (Table 1; S3 Table). The smallest values of *H*_E_ and *H*_O_ were in samples NAD and RIJ, whereas the largest values were in samples SLO and KOT (Table 1; S3 Table). The smallest values of *H*_E_ and *H*_O_ by locus were in Ee2-165b and Ee2-508, respectively, and the largest values were in loci Ej2 and Ee2-507, respectively (Table 2).

**Table 2:** Summary statistics by locus. Total *N*_A_ = observed number of alleles; Mean *N*_A_ = mean number of alleles; *H*_E_ = Expected heterozygosity; *H*_O_ = Observed heterozygosity; *F*_IS_ = coefficient of inbreeding (bold values deviating from HW expectations); *f* Null alleles = frequency of nulle alleles; *F*_ST_ = genetic differentiation estimated.

Locus	Total *N*_A_	Mean *N*_A_	*H*_E_	*H*_O_	*F*_IS_	*f* Null alleles	*F*_*ST*_
Ee2-91b	15	8.7	0.806	0.767	0.048	0.014	0.0012
Ee2-407	38	16.5	0.86	0.805	0.064	0.023	0.0016
Ej41-1	25	11.2	0.69	0.632	**0.084**	0.030	0.0183
Ee10	37	18.5	0.838	0.778	0.072	0.025	0.0619
Ej27.1	36	22.9	0.922	0.869	0.057	0.019	0.0154
Ej35	25	12.2	0.852	0.851	0.001	-0.007	0.0341
Enja83	23	8.7	0.733	0.77	-0.050	-0.029	0.0171
Ee2-507	41	21.5	0.911	0.932	-0.023	-0.020	0.0398
Eja17	15	7.5	0.705	0.614	**0.129**	0.044	0.0096
Ej2	32	20.9	0.943	0.877	0.070	0.027	-0.001
Ee2-135	16	11.5	0.873	0.909	-0.041	-0.026	0.0048
Ee2-165b	12	5.3	0.574	0.558	0.028	0.004	0.0023
Ee2-508	11	6.7	0.672	0.522	**0.223**	0.082	0.0006
Enja148	16	5.3	0.534	0.365	**0.316**	**0.119**	0.0045

Significant departures from the Hardy-Weinberg (HW) proportions over all loci were found in 4 samples, MNA, MNB, BAB and ANC. *F*_IS_ was significant in 4 of 210 tests, and three values of *F*_IS_ indicated homozygote excess (S3 Table). Three loci deviated significantly from HW proportions over all populations (Ej41-1, Eja17 and Ee2-508) (Table 1). Only 4 of 1170 tests (78 pairwise tests X 15 localities) showed significant linkage disequilibrium, which occurred between EJ35 x Ee2-507 and Enja83 x Ee2-507 in the NAD sample, EJ27.1 x EJ35 in the DUG sample and Ee2-507 x Eja17 in the RIJ sample.

### Microsatellite genetic population structure

Overall, genetic differentiation between samples was estimated with 5 statistics. First, pairwise values of Ɵ_ST_ and *F*_ST_ over loci showed similar patterns. Ɵ_ST_ ranged from -0.004 (BAA *vs*. PEB) to 0.054 (NAD *vs*. JAB), and *F*_ST_ ranged from -0.004 to 0.058 between samples. Pairwise Ɵ_ST_ were significant (P < 0.0005) in 51 of 105 comparisons (Table 3). *G*_ST_ ranged from -0.002 (BAA *vs*. PEB) to 0.057 (NAD *vs*. JAB) between samples, *G*'_ST_ ranged from -0.002 to 0.030, and *D*_EST_ values ranged from -0.004 (BAA *vs*. PEB) to 0.194 (NAD *vs*. JAB). Pairwise comparisons were significant (P < 0.0005) in 65 of 105 tests for *G*_ST_ and *G*'_ST_ and in 66 of 105 tests for *D*_EST_ (S4 Table).

**Table 3:** Pairwise multilocus estimates of Ɵ_ST_ (below the diagonal) and *F*_ST_ (above the diagonal). Significant pairwise tests are in bold type.

	MNA	MNB	SLO	NAD	BAA	BAB	KOT	ANC	DUG	JAB	RIJ	PEA	PEB	SPE	CDG
MNA		0.010	0.015	0.033	0.000	0.003	0.009	0.005	0.020	0.026	0.018	0.009	0.003	0.005	0.001
MNB	0.009		0.010	0.035	0.013	0.009	0.007	0.005	0.035	0.037	0.017	0.006	0.017	0.023	0.018
SLO	0.014	0.010		0.037	0.016	0.009	0.004	0.013	0.041	0.047	0.021	0.005	0.019	0.030	0.030
NAD	**0.030**	**0.035**	0.035		0.026	0.020	0.035	0.032	0.051	0.058	0.041	0.030	0.031	0.038	0.029
BAA	-0.002	0.012	**0.015**	**0.023**		0.003	0.013	0.005	0.013	0.018	0.015	0.010	-0.004	0.005	0.000
BAB	0.002	**0.010**	0.009	**0.019**	0.002		0.005	0.003	0.021	0.028	0.013	0.003	0.005	0.008	0.007
KOT	0.009	0.006	0.005	**0.033**	0.011	0.005		0.008	0.026	0.031	0.011	0.002	0.016	0.022	0.017
ANC	0.003	0.004	0.012	0.028	0.004	0.003	0.007		0.019	0.026	0.016	0.007	0.005	0.014	0.009
DUG	0.018	**0.036**	**0.038**	**0.046**	0.011	**0.020**	**0.025**	0.017		-0.002	0.015	0.032	0.016	0.024	0.022
JAB	**0.025**	**0.038**	**0.046**	**0.054**	0.016	**0.027**	**0.031**	0.026	-0.002		0.013	0.037	0.023	0.027	0.028
RIJ	0.017	0.017	**0.020**	**0.038**	0.014	0.011	0.010	**0.016**	**0.016**	0.016		0.016	0.020	0.026	0.021
PEA	0.008	0.005	**0.004**	**0.029**	**0.009**	0.004	0.002	0.006	**0.031**	**0.038**	**0.015**		0.002	0.010	0.014
PEB	0.002	**0.017**	**0.020**	**0.028**	-0.004	0.004	**0.015**	0.004	**0.015**	**0.022**	**0.019**	**0.017**		0.008	0.006
SPE	0.003	**0.022**	**0.028**	**0.035**	0.003	0.007	**0.020**	**0.011**	**0.023**	**0.026**	0.024	**0.017**	0.007		0.000
CDG	-0.001	0.017	**0.029**	**0.026**	-0.002	0.006	**0.016**	0.006	**0.020**	**0.027**	**0.020**	**0.014**	0.006	-0.002	

**Fig 2:**
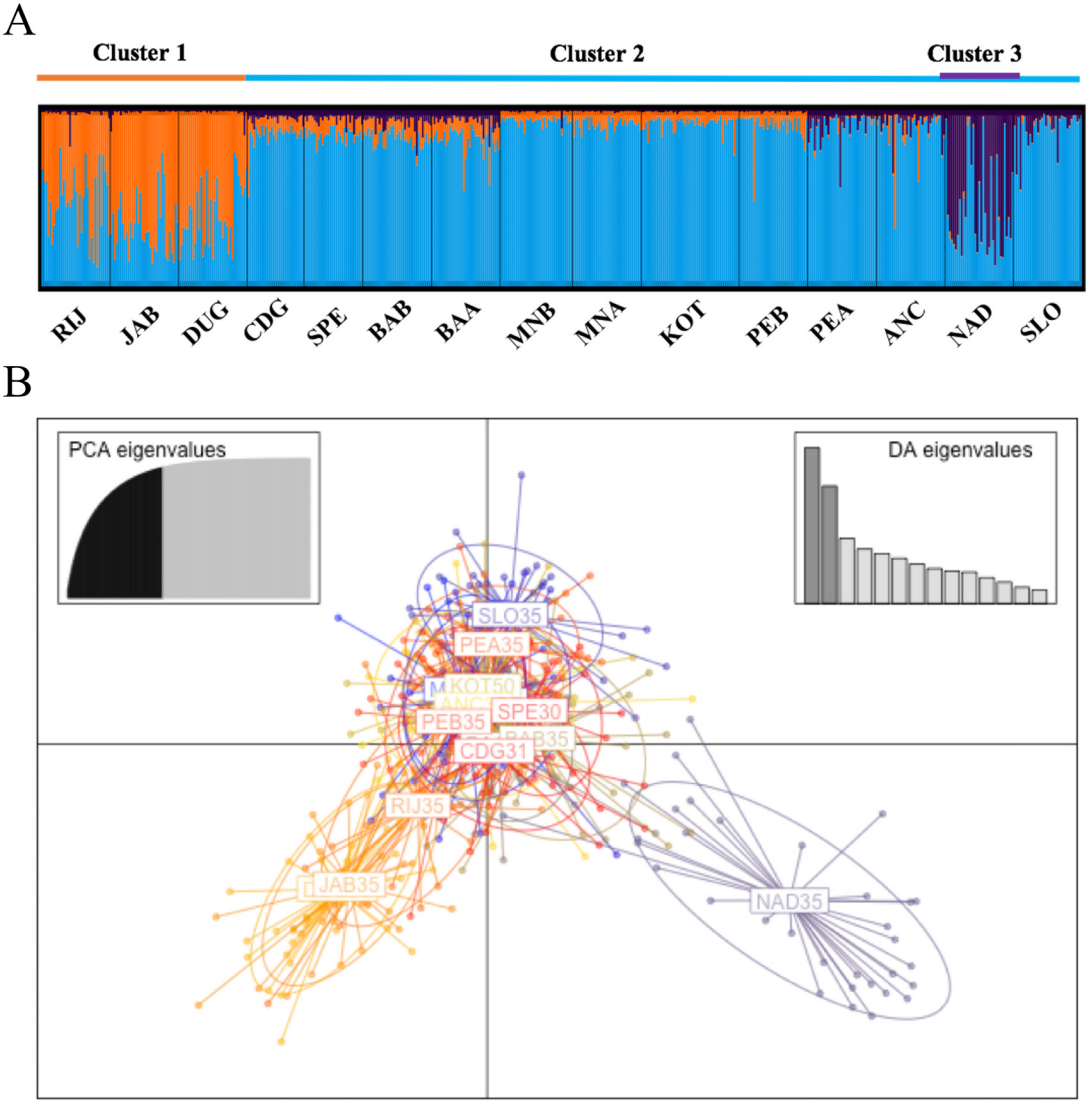
Graphical outcomes of STRUCTURE simulations for *K* = 3 using all loci and the LocPrior function (barplots represent the individual *q* values) (Fig 2A). Graphical outcome of DAPC plot (Fig 2B).

STRUCTURE simulations of neutral loci, with and without LocPrior information, yielded two major clusters (S1A,S1B and S1E Fig): i) DUG+JAB+RIJ (Northeast Adriatic samples) and ii) the remaining samples. However, a high probability of three major clusters emerged using only candidate outlier loci without the LocPrior information (S1C Fig) and using all loci with the LocPrior function (Fig 2A; S1D Fig). These three clusters were largely represented by i) DUG+JAB+RIJ (Northeast Adriatic samples), ii) NAD (Northern Adriatic sample) and iii) the remaining samples. Other possible substructures yielding 5 and 8 clusters appeared in the simulations, but with low *q* values (S1A–S1F Fig).

In the DAPC analysis, the first 2 of the 150 Discriminant Axes (DA) were significant. These DA's defined three major genetic Clusters: i) DUG+JAB+RIJ (Northeast Adriatic samples), ii) NAD (Northern Adriatic sample) and iii) all remaining samples (Fig 2B). The first DA separated mainly the Northeast Adriatic Cluster from the NAD Cluster, while the second DA largely separated those two Clusters from a third, which included the remaining samples (Fig 2B).

A total of 38 of 531 individuals were identified as first generation migrants. The analysis of migration rates and first generation migrants between clusters revealed a higher intra-cluster migration than inter-cluster migration in the two or three genetic groups. The directionality of gene flow was largely from populations MNA and ANC (S5A Table). Estimates of Θ from the historical gene flow ranged between 0.003 in CDG to 0.046 in JAB (S5B Table). Migration rates (*M*) ranged between 18.50 (*M*_JAB→NAD_) and 944.01 (*M*_DUG→PEA_). The directionality of historical gene flow suggested a pattern that was similar to sea-surface circulation (Fig 1B). The main sources of migrants were DUG, ANC and CDG (S5C Table).

Additional approaches resolved other features of geographic structure. First, BARRIER identified at least three major allele-frequency discontinuities, indicating a heterogeneous pattern of partial isolation between locations. These results indicated two strong barriers, one between NAD and nearby sampling areas (Fig 1B, barrier a, dotted line) and a longitudinal barrier across the Mid Adriatic Pit that divided Adriatic anchovies into east-west groups (Fig 1B, barrier b, solid line). In addition, a north-south barrier that divide the Mid Adriatic Pit was detected (Fig 1B, barrier c, dashed line). This barrier was stronger in the east than in the west.

Even though differences appeared between populations, no isolation-by-distance over the length of the Adriatic was detected with Mantel's test between pairwise ϴ_ST_ and geographical distances (*P* > 0.05; a = 0.0165172, b = 0.00000108) (data not shown).

### Outlier loci and environment-allele frequency correlations

The methods used to identify outlier loci showed slightly different results, but together identified five candidate outlier loci among the 13 analyzed, including Ee2-91b, Ee2-407, Ee10, Ee2-507 and EJ2 (Table 4). Fdist in Lositan identified seven outlier loci: three candidate loci for directional selection (Ee10, EJ35, Ee2-507) and four for balancing selection (Ee2-91b, Ee2-407, EJ2 and Ee2-135) (S2 Fig; Table 4). BayeScan indicated highly significant probabilities for outlier candidate loci for balancing selection (negative alpha values) for all loci, with the exception of EJ35 (*P* > 0.05) (Table 4), but *q* values were under the 10% threshold for FDR (False Discovery Rate) correction. The Hierarchical Island Method (HIM) for clustering in STRUCTURE *K* = 2 postulated six outliers, with two loci (Ee10 and Ee2-507) as candidates for directional selection and four loci (Ee2-91b, Ee2-407, EJ2 and Ee2-165b) for balancing selection (S3A Fig; Table 4). With *K* = 3, HIM indicated that five of these six loci (except Ee2-165b) remained significant, showing the same pattern of selection as tests with *K* = 2 (S3B Fig; Table 4). Finally, the LnRH test yielded 42 of 1365 pairwise combinations of locations and loci with values over the ±2.58 threshold. These tests were associated to two loci: Ee2-507 for 31 tests and EJ27.1 for 11 tests (S6 Table).

**Table 4:** Observed *F*_ST_ values from Lositan [55, 56], BayeScan [57] (plus *q* values) and the Hierarchical Island Model (HIM) [58] to test for non-neutral loci.

Locus name	Obs *F*_ST_ Lositan	Obs *F*_ST_ BayeScan	Obs *F*_ST_ HIM K = 2	Obs *F*_ST_ HIM K = 3
**Ee2-91b**	0.0026**	0.0130*** (0.000)	0.0021*	0.0017**
**Ee2-407**	0.0023***	0.0033*** (0.000)	0.0016*	0.0015**
Ej41-1	0.0216	0.0164*** (0.000)	0.0195	0.0233
**Ee10**	0.0661***	0.0269*** (0.000)	0.0698**	0.0659***
Ej27.1	0.0167	0.0103*** (0.000)	0.0400	0.0293
Ej35	0.0343**	0.0300 (0.000)	0.0448	0.0427
Enja83	0.0163	0.0205*** (0.000)	0.0299	0.0243
**Ee2-507**	0.0397***	0.0193*** (0.000)	0.0771***	0.0623***
Eja17	0.0111	0.0152*** (0.000)	0.0250	0.0162
**Ej2**	0.0001*	0.0027*** (0.000)	0.0003**	0.0004*
Ee2-135	0.0046**	0.0062*** (0.000)	0.0039	0.0040*
Ee2-165b	0.0036	0.0084*** (0.000)	0.0018*	0.0080
Ee2-508	0.0059	0.0185*** (0.000)	0.0099	0.0044

Level of significances:

* P<0.05;

** P<0.01;

*** P<0.001.

Loci showed in bold type represent those resulted significant in all selection tests.

**Fig 3:**
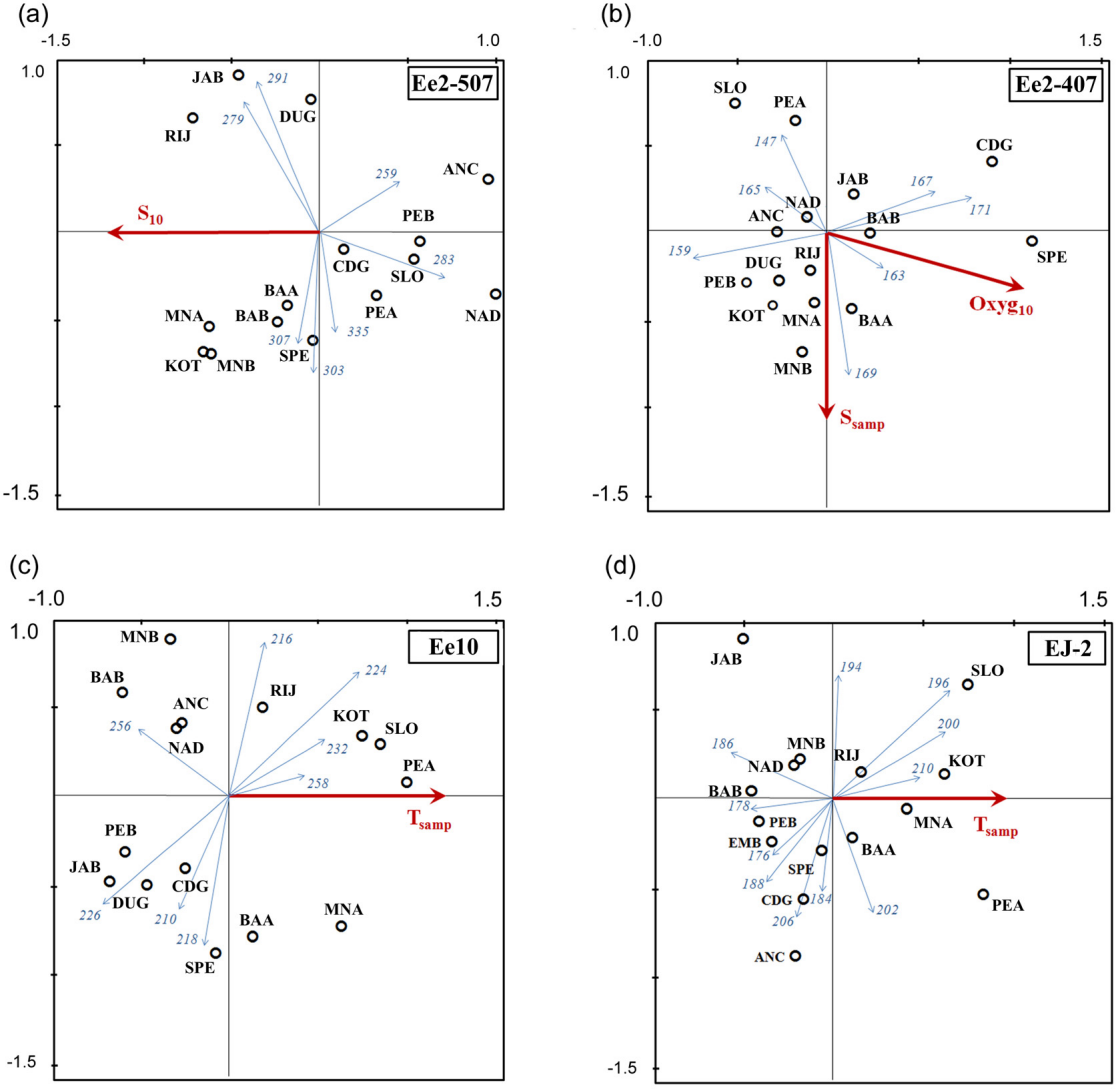
Redundancy Discriminant Analysis (RDA) triplots. Triplots show the distribution of samples (circles) relative to major significant environmental variables (dashed arrows) and microsatellite allele frequencies (solid arrows) at four candidate outlier loci. Triplots show results for Ee2-507 (A), Ee2-407 (B), Ee10 (C) and EJ2 (D), respectively.

The Redundancy Discriminant Analysis (RDA) identified significant correlations between allelic frequencies at four outlier loci (Ee2-507, Ee2-407, Ee10 and EJ2) and four environmental variables, including temperature at sampling depth (*T*_samp_), salinity at sampling depth (*S*_samp_) and at 10 m (*S*_10_) and dissolved oxygen concentration at 10 m (*Oxyg*_10_). At Ee2-507, alleles 279 and 291 were positively correlated and alleles 259 and 283 were inversely correlated with S_10_ (both *P* < 0.019) (Fig 3A). Significant correlations appeared for Ee2-407 with both *Oxyg*_10_ and *S*_samp_ (*P* < 0.002); allele 169 varied positively and allele 147 inversely with *S*_samp_, and alleles 163 and 171 were positively and allele 159 was inversely correlated with *Oxyg*_10_ (Fig 3B). At locus Ee10, alleles 224, 232 and 258 were positively and alleles 226 and 256 were inversely correlated with *T*_samp_ (*P* < 0.003) (Fig 3C). Finally, at EJ2, alleles 196, 200 and 210 were positively and alleles 178 and 186 were inversely correlated with *T*_samp_ (P < 0.033) (Fig 3D).

**Fig 4:**
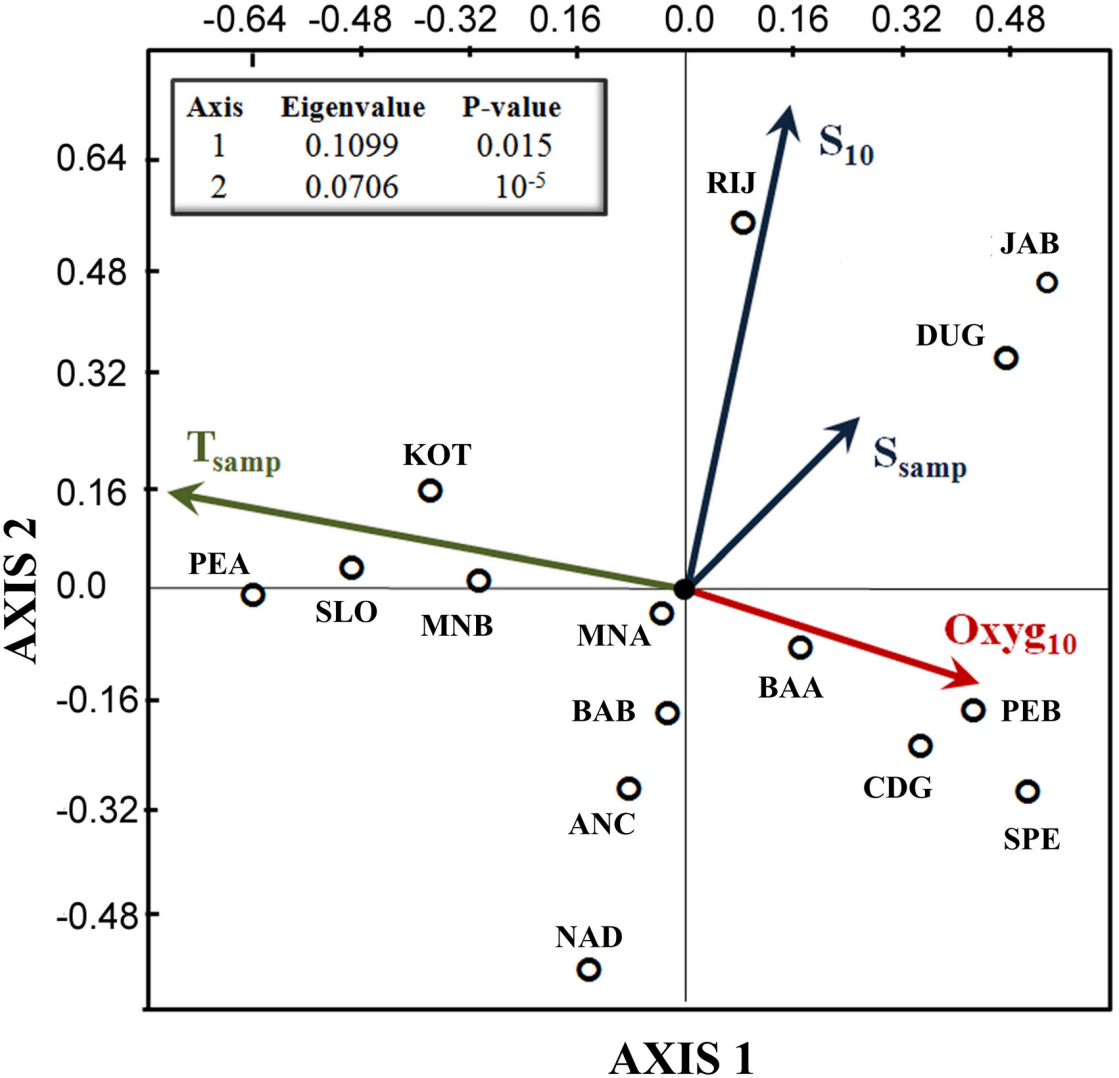
Canonical Correspondence Analysis (CCA) of microsatellites and environmental variability. The plot shows the distribution of samples with candidate outlier loci that were correlated with the four environmental variables, *T*_samp_, *S*_10_, *S*_samp_, *Oxyg*_10_. Arrows identify the four environmental variables and their direction explain which samples are most correlated with them.

Both the first and second axes of the CCA ordination of samples with four environmental variables were significant (*P* < 0.05 and *P* < 0.001, respectively). The results indicated a positive association for allele frequencies in EMB, EMC and EMD and a negative association for allele-frequencies in sample EMA with salinity *S*_samp_ and *S*_10_ (Fig 4). Samples SLO, KOT and PEA were positively and samples SPE and CDG from the Tyrrhenian Sea were negatively associated with temperature at sampling depth (*T*_samp_). Finally, SPE and CDG were directly associated with dissolved oxygen content in the first 10 m (*Oxyg*_10_) and inversely associated with *T*_samp_.

### Detection of multiple species of anchovies in the Adriatic Sea

The STRUCTURE analysis using the LocPrior function to cluster sampling sites by bathymetry showed three genetic clusters, as in the other STRUCTURE analyses (S1G Fig). In general, the pattern did not show a clear association with bathymetry as postulated by Borsa *et al*. [21]. Furthermore, the AMOVA test partitioned by ocean depth of 50 m, indicated that the largest variance was among individuals within populations (97.86% of total variance; S7 Table). Although variation among populations within groups (*F*_SC_ = 0.0017) was significant (P < 0.05), it explained only a small amount of the total variation (1.79%). Divergence among groups (*F*_CT_ = 0.0012) was not significantly different from zero (*P* > 0.05).

## Discussion

The results of our survey of microsatellite DNA variability among populations show that while anchovies in the Adriatic are genetically subdivided among regions to some extent, none of these populations represent separate species, as previously proposed. The mechanisms producing divergence between populations include both demographic processes influenced by ocean currents and fronts and natural selection that leads to locally adapted populations. Before exploring these factors in more detail, we offer the following evaluation of the study. While the sample sizes used in this study were not large, they provided sufficient statistical power to detect population differences. Statistical power was enhanced by the use of a large number of microsatellite loci encoded in nuclear genes. These markers provided complementary insights into population structure and local adaptation. We additionally used several approaches to the analyses of the microsatellite data that illuminated various dimensions of population structure.

### How many anchovy species in the Adriatic Sea?

Borsa [63] and Borsa *et al*. [21] postulated that two species of anchovy inhabit the Adriatic Sea on the basis of north-south allele-frequency gradients reported by Bembo *et al*. [22] and on morphological parallels with two putative anchovy species in the Gulf of Lyon [21, 63]. The recently described estuarine species, *E*. *albidus* Borsa, Collet et Durand, 2004, resembled shallow-water anchovies in low-saline areas of the northern Adriatic [21]. The larger, bluish anchovies in the Adriatic resembled European anchovies elsewhere in open, deep waters with salinities greater than 38 psu, and were postulated to be *E*. *encrasicolus* [21]. However, our analysis of nuclear markers in anchovies from throughout the Adriatic did not show genetic differentiation between coastal and deep-water populations that could be interpreted to indicate multiple species.

Nevertheless, it might be argued that the genetic differences in the northern Adriatic Sea support the occurrence of *E*. *albidus* in the low-saline plume of the Po River (NAD). Although these anchovies share some ecological and morphological traits with putative *E*. *albidus* in the Gulf of Lyon, Po River anchovies are genetically most similar to oceanic anchovies in the central Adriatic. The level of genetic differentiation between NAD and the other samples suggests a substantial genetic separation, but this level of divergence is not large enough to indicate the presence of two divergent species. The color and morphological variability observed among anchovy populations in the Adriatic Sea (and in the Gulf of Lyon) likely reflects plastic phenotypic responses to environmental variability in anchovies that are typical of anchovies in the Mediterranean Sea [64].

### Genetic variability among populations

The small values of *F*_ST_ and ϴ_ST_ between Adriatic populations are typical of divergence between other population groups of anchovies or between populations of other pelagic fishes. The overall levels of genetic differentiation between populations in the Adriatic Sea were similar to those between anchovy populations in the eastern Atlantic [65, 66]. As in many marine species, gene flow in Adriatic anchovies occurs by larval dispersal in currents, or by adult movement at low levels over long periods, and prevents the accumulation of genetic differences among populations [67]. Nevertheless, significant differences between some samples indicate that Adriatic anchovies are not entirely panmictic.

The analysis of neutral makers with STRUCTURE resolved two major population groups: populations in northeastern coastal areas (DUG, JAB and RIJ) and the remaining populations in the Adriatic and Tyrrhenian seas. The genetic uniqueness of northeastern anchovies may be due to adaptation to habitats in the numerous coves and embayments and around offshore isles [17, 18, 68]. The northeastern Adriatic appears to be conducive to spawning and early larvae growth and is an area where spawners converge [69].

A north-south genetic discontinuity in the northern Adriatic Sea was previously reported by Bembo *et al*. [22], who found an allozyme-frequency gradient between northern and southern populations. Bembo *et al*. [22] focused on the possible role of depth, defining the 40–50 m sill as the population boundary, rather than on other environmental variables. In our analyses, depth did not appear to be an important isolating variable, as we found no genetic partitioning of microsatellite variability by ocean depth in areas where samples were collected.

Instead, the oceanic front between the Istrian Peninsula and Mid Adriatic Pit may explain, in part, the north-south genetic discontinuity by limiting gene flow. The anticlockwise gyre off the Po River delta may also act as a barrier to gene flow [17, 18]. Although both the front and the gyre are ephemeral, they are strongest in autumn [17, 18] at the end of the anchovy spawning season when larvae and juveniles are most abundant [19, 68]. Larval retention by ocean fronts and gyres is a well-known isolating mechanism in other pelagic fishes [70, 71]. Hence, these oceanic mechanisms may limit the movement of larvae and juveniles and prevent mixing between larvae and juveniles from neighboring spawning sites. The effect of small coastal eddies limiting the dispersals of early life-history stages was also invoked by Borrell *et al*. [66] to explain genetic differences between nearby populations in the Bay of Biscay.

Individual assignment showed that about 10% of the fish in our samples represented first-generation migrants from other populations. However, patterns of dispersals revealed by these tests have to be interpreted with caution, because small samples sizes and finite geographical sampling introduce some randomness into the results. The results also represent only a snapshot of a dynamic process that may change over time. Nevertheless, the results indicate that dispersals between populations are asymmetrical: some populations act as 'sources' and others act as 'sinks'. The extent that populations receiving migrants depend on immigration to persist has to be evaluated with long-term abundance data. With these caveats, most migrants originated from only a few source locations (mostly MNA and ANC samples) and moved to several nearby sink locations following a pattern of dispersion that is similar to major sea-surface currents in the area. The evaluation of historical patterns of gene flow using Migrate-n suggests a similar scenario for both the magnitude and pattern of connectivity. This pattern of migration may also be related to the lack of divergence between Adriatic and Tyrrhenian anchovies because of reciprocal gene flow between the two seas. These results are in line with a recent analysis using SNPs markers showing genetic similarities between Adriatic and Western Mediterranean population of anchovies [72].

### Local adaptation

While the small genetic distances between samples indicate high levels of gene flow, as expected for a pelagic species, the pattern of geographic differentiation and the associations of microsatellite loci with environmental variables indicate that gene flow may be countered by adaptation to local conditions. For example, the timing of spawning, relative to seasonal bursts of productivity can have a strong effect on year-class survival [73, 74]. The persistence of genetic population structure in Adriatic anchovies may indicate differential reproductive success or reduced fitness of migrants reproducing outside their natal spawning areas. If so, adaptation to local habitats explains some of the genetic population structure observed in Adriatic anchovies.

Our inferences about natural selection are based on non-coding microsatellite loci, which are unlikely to be the direct focus of selection, except when microsatellite alleles modify the functions of genes in which they are embedded [75, 76]. Alternatively, microsatellites may show signals of selection when they are linked to genes under selection. The strength of a signal from this 'hitchhiking' effect depends on the strength of selection, recombination rates and genetic drift [77, 78]. Our analyses identified 5 of 13 loci (38.46%) as candidate outliers, and two loci under directional selection (Ee10 and Ee2-507) (15.38%), indicating the potential role of natural selection in driving differentiation among populations. The proportion of candidate outliers was large, but consistent with other studies that showed between 5–15% outlier loci under directional selection [79, 80].

Several life-history traits can interact to facilitate local adaptation. For instance, it is well known that this species has a high skew in reproductive success, which determines the level of genetic diversity in each cohort. Sweepstakes reproduction can promote selection at local scales, especially when coupled with large demographic variation in census size. During population bottlenecks, the reduction in effective population size (*N*_e_) can produce transient genetic sweeps that lead to locally adaptive genotypes [81]. A recent study showed that the effective sizes of anchovy populations in the Adriatic are several orders of magnitude smaller than census sizes [82]. These small effective sizes appear to be in part a legacy of a severe reduction in population abundances from overharvesting in the 1980s. Small effective population sizes in anchovies suggest that genetic drift, together with repeated selective sweeps, can mold the anchovy genome, resulting in local adaptation to major environmental drivers.

To further investigate the molecular footprints of selection, we tested candidate outlier loci for associations with environmental variables suspected to be important in the physiologies and ecologies of local anchovy populations. Four of the five candidate outliers were significantly correlated with environmental variables. Interestingly, these variables, especially salinity and temperature, influence larval development, survival and growth [19, 68], as well as, adult maturation and reproductive success [83]. Associations with salinity and temperature variation explained patterns of genetic variation. The northeastern group of anchovies showed a positive association with higher salinities, whereas anchovies in the Po River Plume showed an inverse association with salinity. The waters in these adjoining areas showed the largest difference in salinity among the sites sampled in our study, with the Po River plume having the lowest salinity, and waters of the eastern Adriatic along the northern Croatian coast having the highest salinities in the whole Adriatic Sea [17, 18]. Natural selection from strong differences in salinity among some areas may explain the genetic divergence between populations in the northern Adriatic Sea, despite substantial levels of gene flow between these areas.

In the CCA analysis, a set of samples from shallow Adriatic coastal waters (SLO, KOT and PEA) were positively associated with temperature variability, whereas samples from the Tyrrhenian Sea (SPE and CDG) were inversely associated with temperature variability. This is consistent with high seasonal fluctuations in temperature in northern and western Adriatic coastal areas, in contrast to greater thermal stability in the Tyrrhenian basin [68, 84]. Temperature also appears to be an important selective factor among populations of anchovies in the Eastern Atlantic [85].

Adriatic anchovies are also influenced by variability in dissolved oxygen, which is inversely correlated with temperature, because oxygen saturation in water decreases at higher temperatures. Populations in the Tyrrhenian Sea (SPE and CDG) were positively associated with dissolved oxygen content, but coastal populations in shallow waters (SLO, KOT and PEA) were inversely associated with oxygen content. The dissolved oxygen content may interact with temperature to define local selective constraints. Higher water temperatures in summer generally lead to a drop in dissolved oxygen, producing hypoxic and anoxic conditions [86], especially in the Adriatic Sea.

Genetic discontinuities among populations of anchovies may be related to the effects of environmental variables on the growth and survival of early developmental stages, as well as on adult reproductive success. The complex genetic patterns among populations of marine organisms have only recently been correlated with the influence of environmental variables on the genomes of several marine species [11]. Numerous studies show that environmental variables affect adaptive responses in marine fishes on local (European hake [15]) and oceanic scales (Atlantic anchovies [85]; Bluefin Tuna [14]).

### Conclusions

An important result of this study indicates that anchovies throughout the Adriatic belong to a single species, *E*. *encrasicolus*. Nevertheless, the individuals collected in the study area, which covered the entire Adriatic basin and part of the Tyrrhenian Sea, were partitioned into two major populations which were sometimes further sub-structured. Adriatic anchovy subpopulations show a patchy distribution of genetic variability that appears to reflect selective processes mediated by several environmental variables, including salinity, temperature and dissolved oxygen content. The relationship between environmental variables and the distribution of genetic differences among population groups is consistent with adaptation to local environmental conditions. Salinity, in particular, appears to be important in structuring northeastern Adriatic anchovy populations. Our geographical survey of microsatellite DNA variability reveals a more complex genetic structure among Adriatic anchovy populations than was previously detected, but does not confirm the presence of a second species of *Engraulis*. Further resolution of this biocomplexity will provide a basis for understanding adaptive mechanisms and a basis for optimizing management strategies for sustainable harvests.

## Supporting Information

S1 Fig Graphical plotting of simulations carried out using STRUCTURE [43, 44] under various settings. The simulations were run using i) all 13 loci, ii) only neutral loci and iii) only candidate outliers with and without the assistance of prior information (S1A–S1F Fig). Each simulation shows the mean logarithmic probability with standard deviation (SD) obtained for each K tested (1 < *K* < 10; figure on the left) and the delta *K* probability obtained after the use of Evanno method [48] (2 < *K* < 9; figure on the right). Additionally, a simulation was carried out dividing sampling sites based on bathymetry using a threshold of 50 m to detect population structure associate with potential occurrence of different anchovy species in the covered area (S1G Fig).
(DOCX)

S2 Fig Graphical plot of fdist/Lositan [55, 56] simulation. Blue dots represent interpolation between mean values of *F*_ST_ and *H*_E_ in each marker (microsatellite locus) analyzed. Blue dots falling within light grey area represent neutral markers whereas those within the yellow and the red areas represent outlier markers under balancing and directional selection, respectively.
(DOCX)

S3 Fig Graphical plot of Hierarchical Island Model [59] simulations under the assumption of population structured in two (*K* = 2; S3A Fig) and three (*K* = 3; S3B Fig) clusters. Dots represent the interpolation between mean values of *F*_ST_ and *H*_E_ in each marker (microsatellite locus) analyzed. Red, green and purple lines represent 1%, 5% and 95% percentiles of *F*_ST_ null distribution, respectively.
(DOCX)

S1 File Contains information concerning the PCR multiplexing protocols, the genotyping procedures and the power analysis with different set of markers to detect genetic differentiation. (DOCX)

S1 Table Summary of the microsatellite loci and primers for their PCR amplification used in the present study. (DOCX)

S2 Table Values of the 13 environmental variables considered for each locality. (DOCX)

S3 Table Summary of genetic variability observed at 14 microsatellite loci in the sampled locations. (DOCX)

S4 Table Pairwise comparisons of several differentiation genetics estimators. In S4A Table are showed values from *G*'_ST_ and *G*_ST_ while in S4B Table the values from *D*_EST_.
(DOCX)

S5 Table In S5A Table are provided results from the assignment test for first generation migrants. The numbers within the table refer to the individuals that were allocated outside the sampling localities examined in this study (receiving sampling locations). The original sampling sites detected for each migrant are reported along the first row (native sampling locations). The S5B Table provide values for the demographic estimator Theta (Θ), while S5C Table shows migration rates (M) of historical gene flow. The sampling location in the first line represent the source locations while the sampling location in the first column the receiving locations.
(DOCX)

S6 Table Pairwise estimates of LnRH values [60] for detecting loci under selection. Values in red are those exceeding the threshold of significance set at ±2.58.
(DOCX)

S7 Table AMOVA on microsatellite DNA. Samples were divided into groups according to the bottom depth.
(DOCX)
