# Supplementary material for: Biocomplexity in Populations of European Anchovy in the Adriatic Sea
Source: PLoS One. 2016 Apr 13;11(4):e0153061. doi: 10.1371/journal.pone.0153061 (PMC4830579; doi:10.1371/journal.pone.0153061)

S1_A Fig

**STRUCTURE_Simulation based on the use of 13 loci (neutral plus candidate outliers) WITHOUT the LocPrior function. a) Plot of values for simulated mean Logarithmic Probabilities from each K. b) Plot of best K based on Evanno method. c) Barplots of best K outcomes.**

1. **b)**

**c) K = 2 (above); K = 6 (below)**


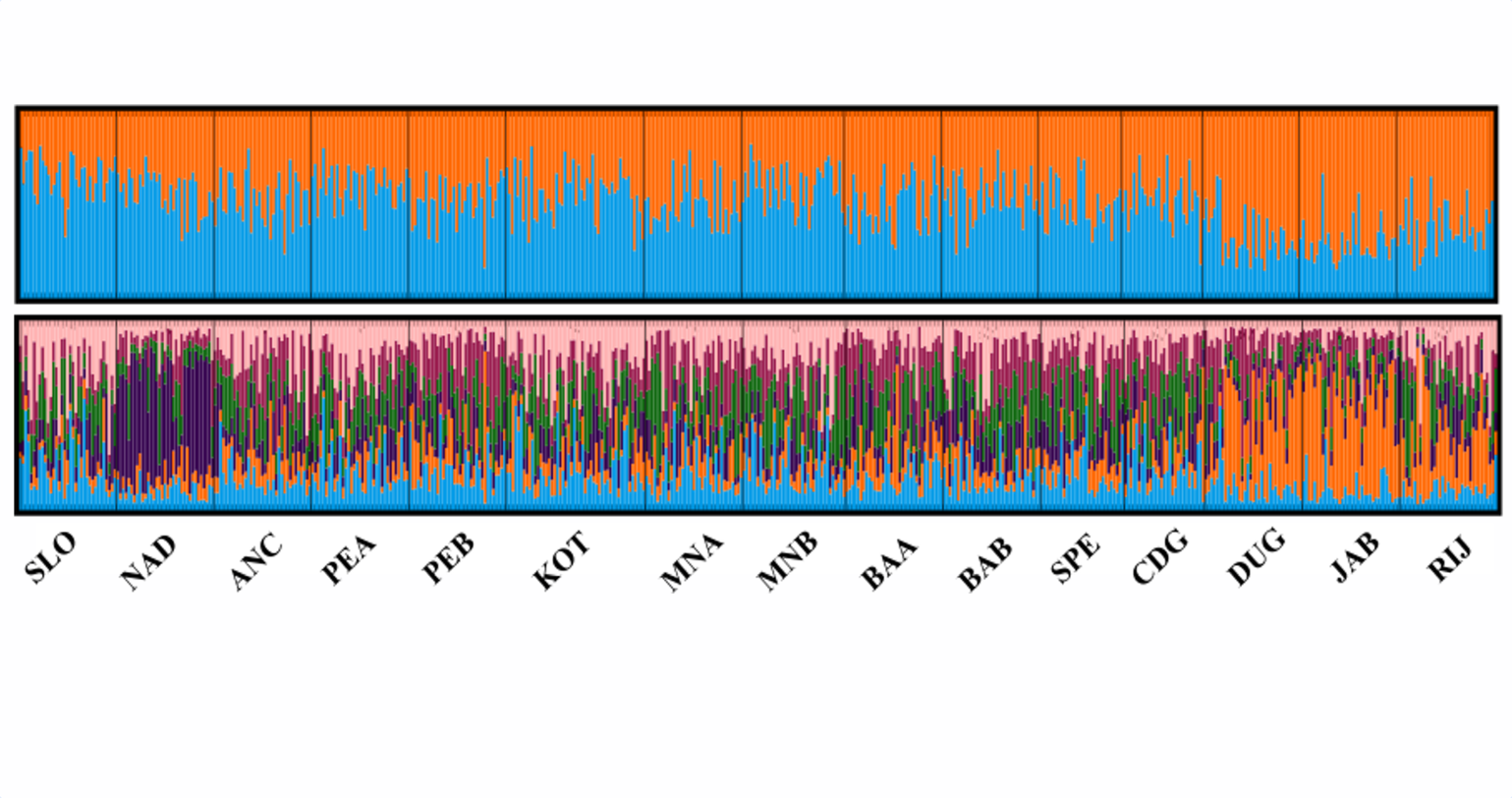


S1_B Fig

**STRUCTURE_Simulation based on the use of 9 neutral loci WITHOUT the LocPrior function. a) Plot of values for simulated mean Logarithmic Probabilities from each K. b) Plot of best K based on Evanno method. c) Barplots of best K outcomes.**

1. **b)**

**c) K = 2 (above); K = 6 (below)**


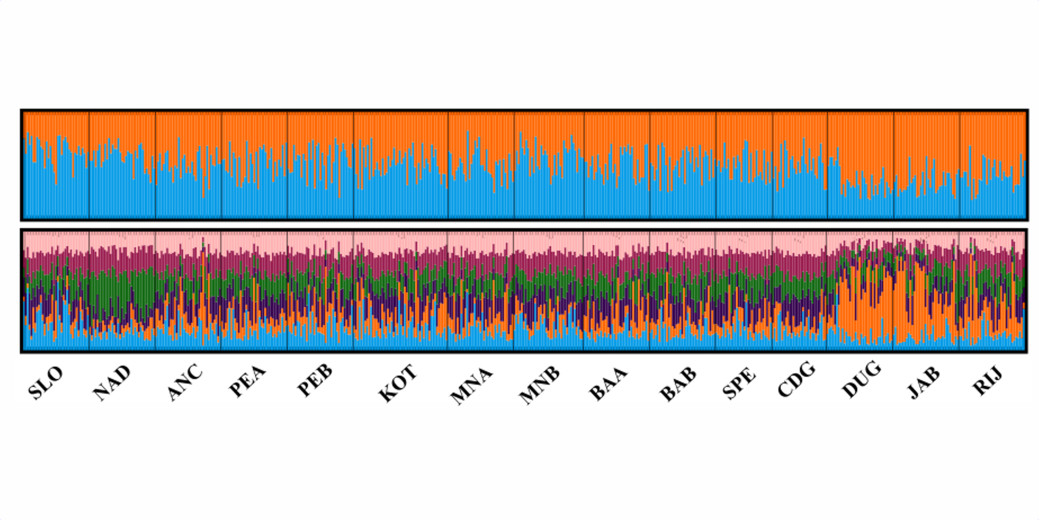


S1_C Fig

**STRUCTURE_Simulation based on the use of 4 candidate outlier loci WITHOUT the LocPrior function.**

1. **b)**

**c) K = 3 (above); K = 6 (below)**


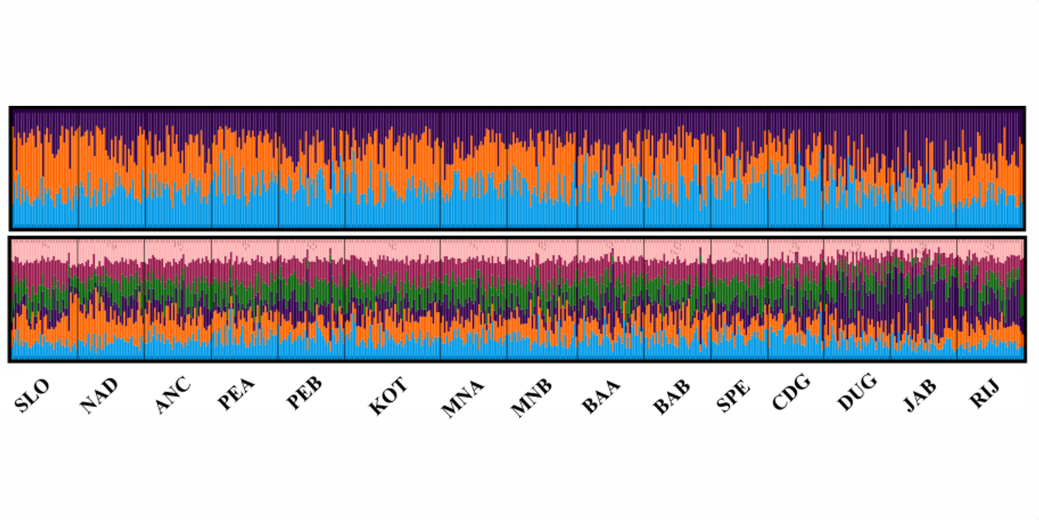


S1_D Fig

**STRUCTURE_Simulation based on the use of 13 loci (neutral plus candidate outliers) WITH the LocPrior function. a) Plot of values for simulated mean Logarithmic Probabilities from each K. b) Plot of best K based on Evanno method. c) Barplots of best K outcomes.**

1. **
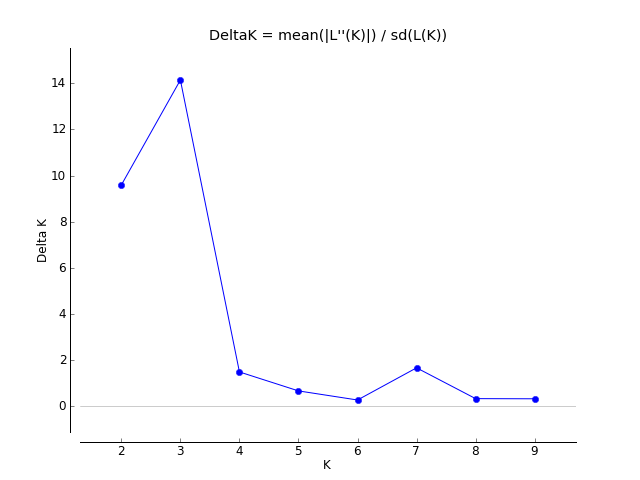

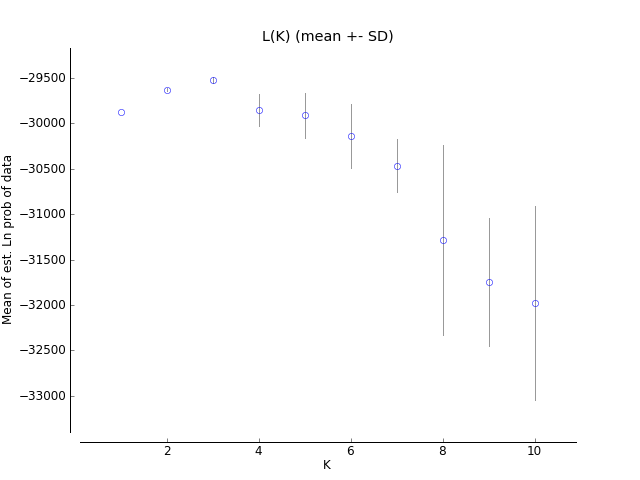
 b)**

**c) K = 3 (above); K = 7 (below)**


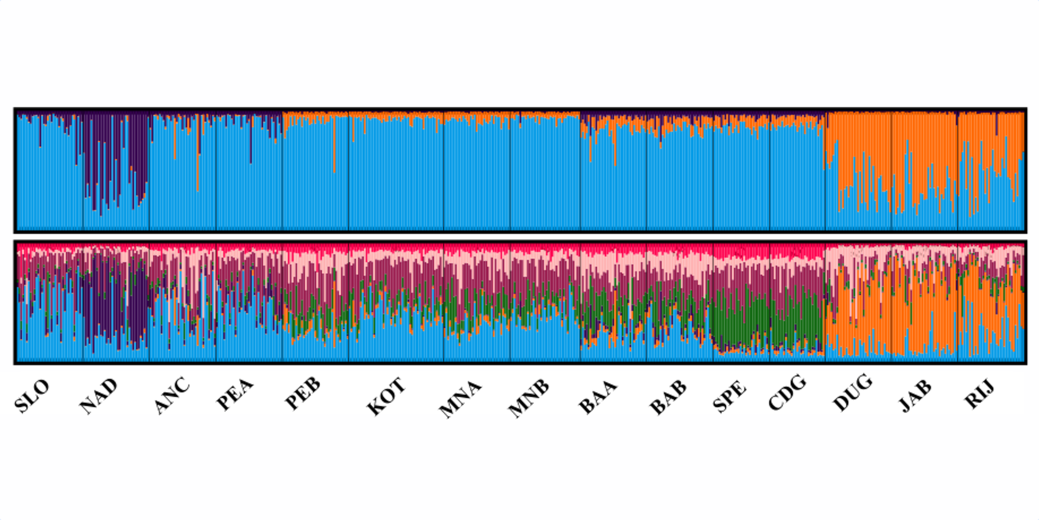


S1_E Fig

**STRUCTURE_Simulation based on the use of 9 neutral loci WITH the LocPrior function. a) Plot of values for simulated mean Logarithmic Probabilities from each K. b) Plot of best K based on Evanno method. c) Barplots of best K outcomes.**

1. **b)**

**c) K = 2 (above); K = 5 (middle); K = 8 (below)**


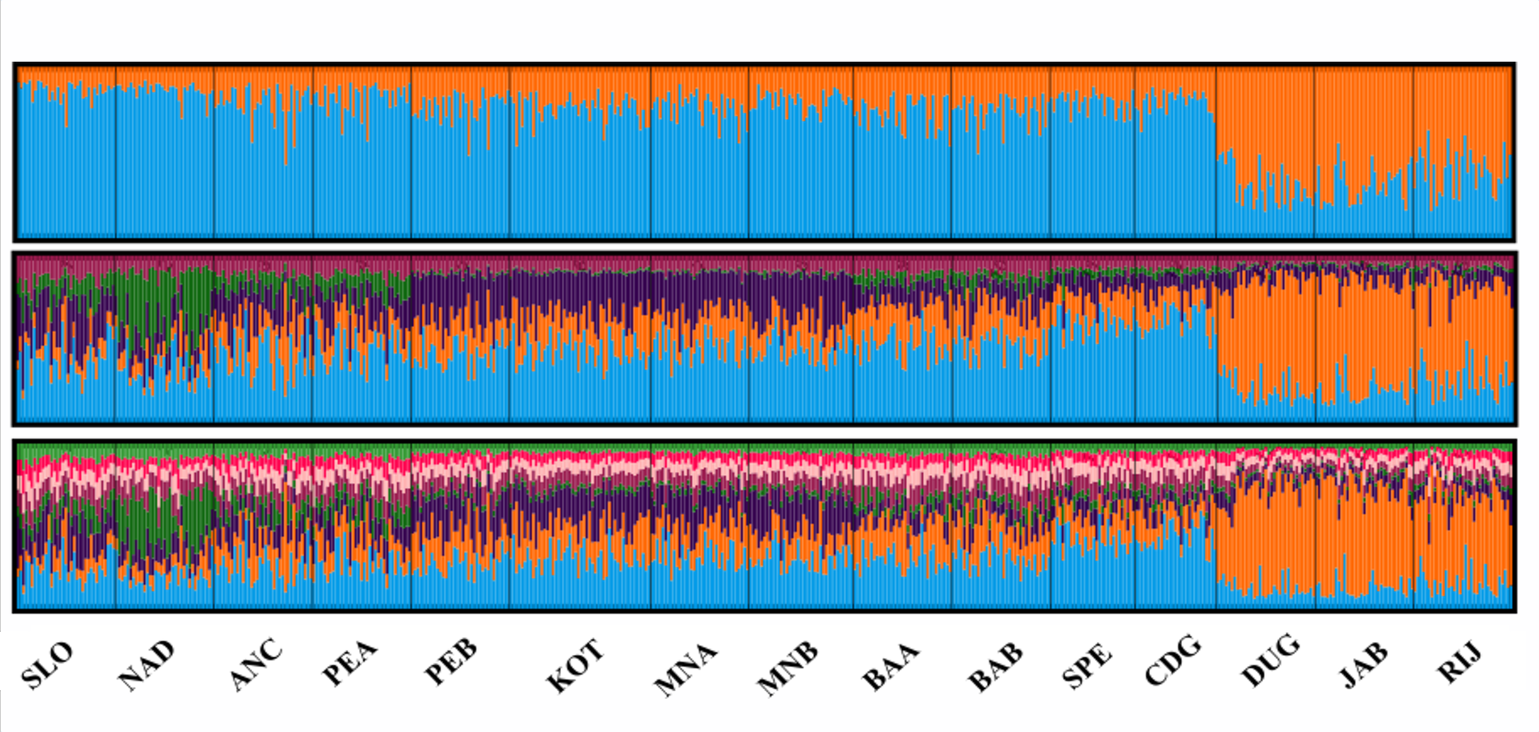


S1_F Fig

**STRUCTURE_Simulation based on the use of 4 candidate outlier loci WITH the LocPrior function. a) Plot of values for simulated mean Logarithmic Probabilities from each K. b) Plot of best K based on Evanno method. c) Barplots of best K outcomes.**

1. **b)**

**c) K = 2 (above); K = 4 (below)**


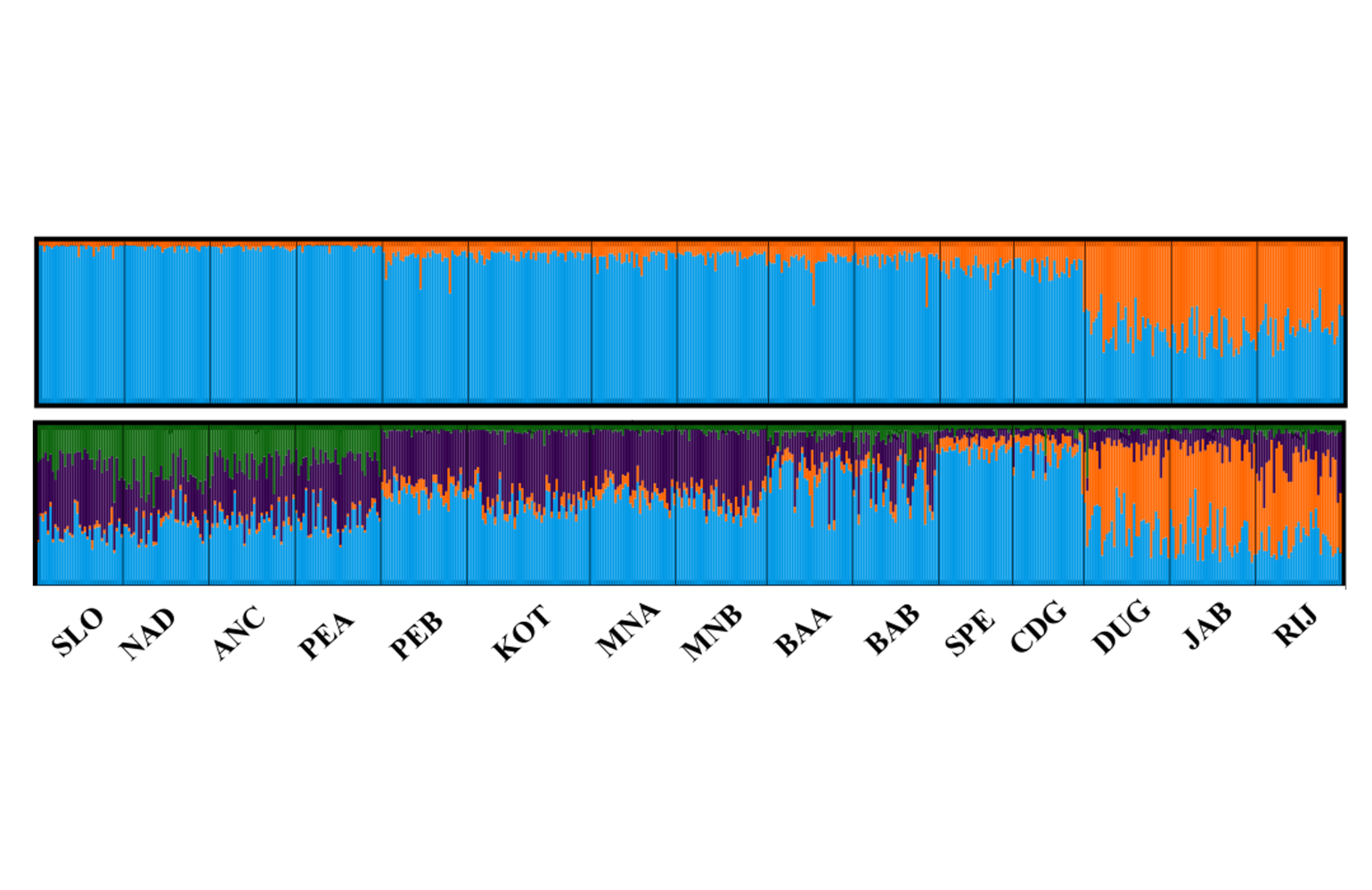


S1_G Fig

**STRUCTURE_Simulation based on the use of all candidate outlier loci WITH the LocPrior function used to group sampling locations based on the bathymetry threshold of 40m. a) Plot of values for simulated mean Logarithmic Probabilities from each K. b) Plot of best K based on Evanno method. c) Barplots of best K outcomes. d) Barplot of K=2 outocome under the scenario of different bathymetry clustering.**

1. **b)**

**c) K = 3 (above); K = 5 (middle); K = 7 (below)**


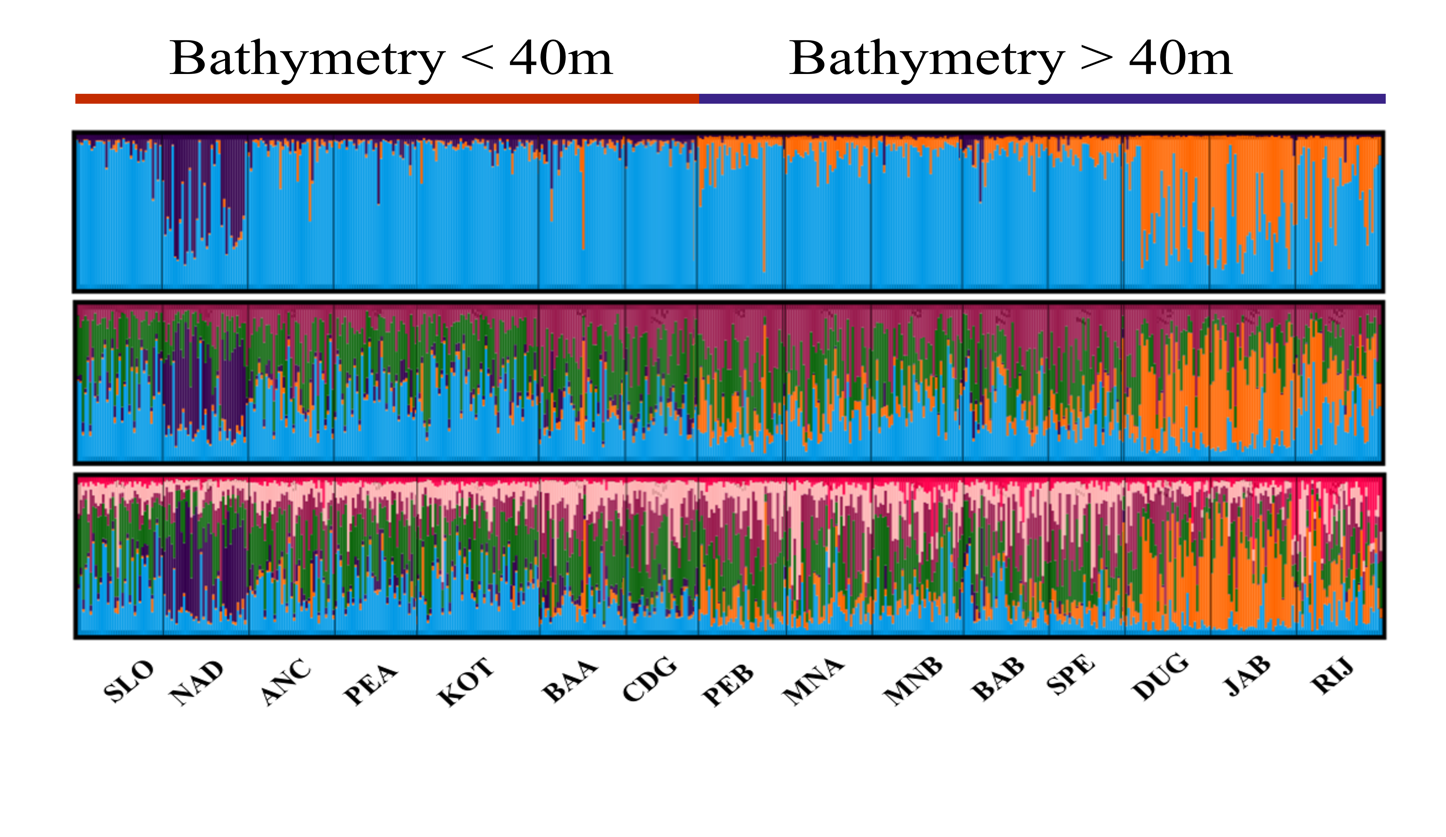


1. **K = 2**


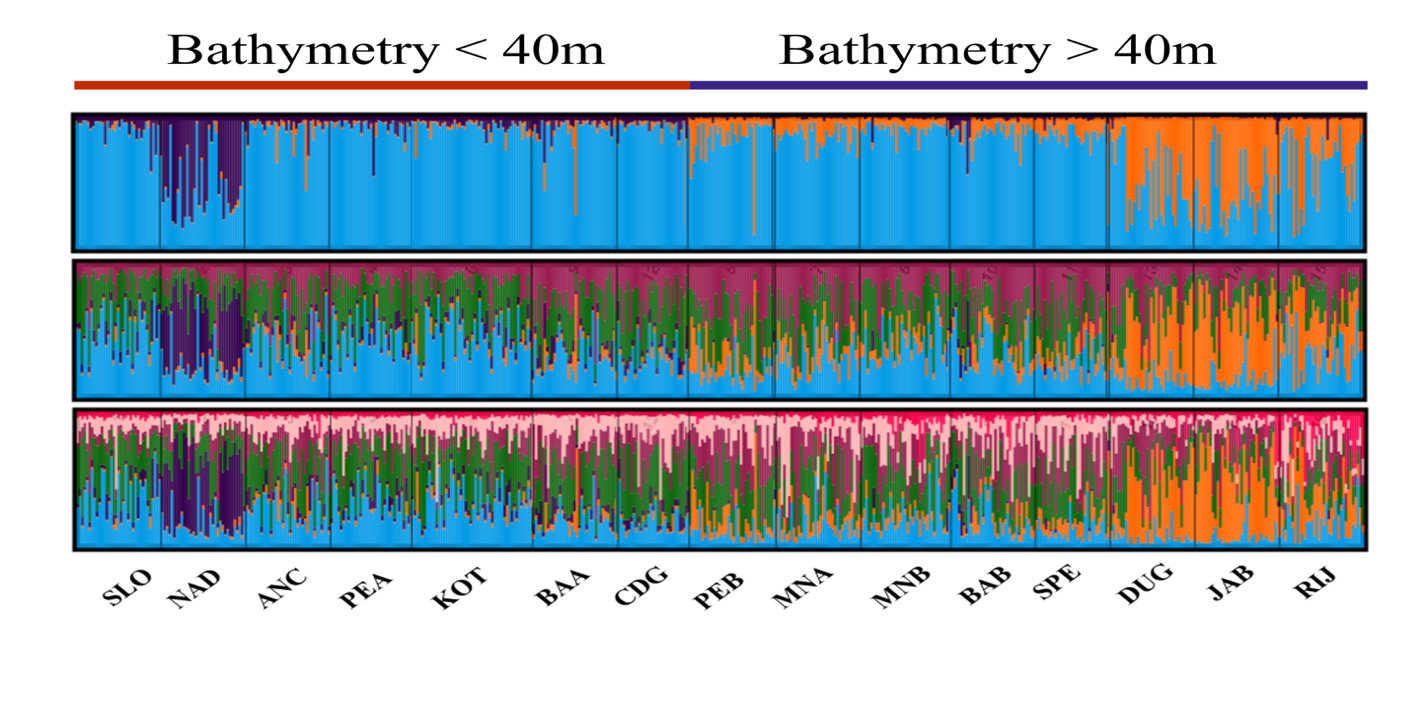

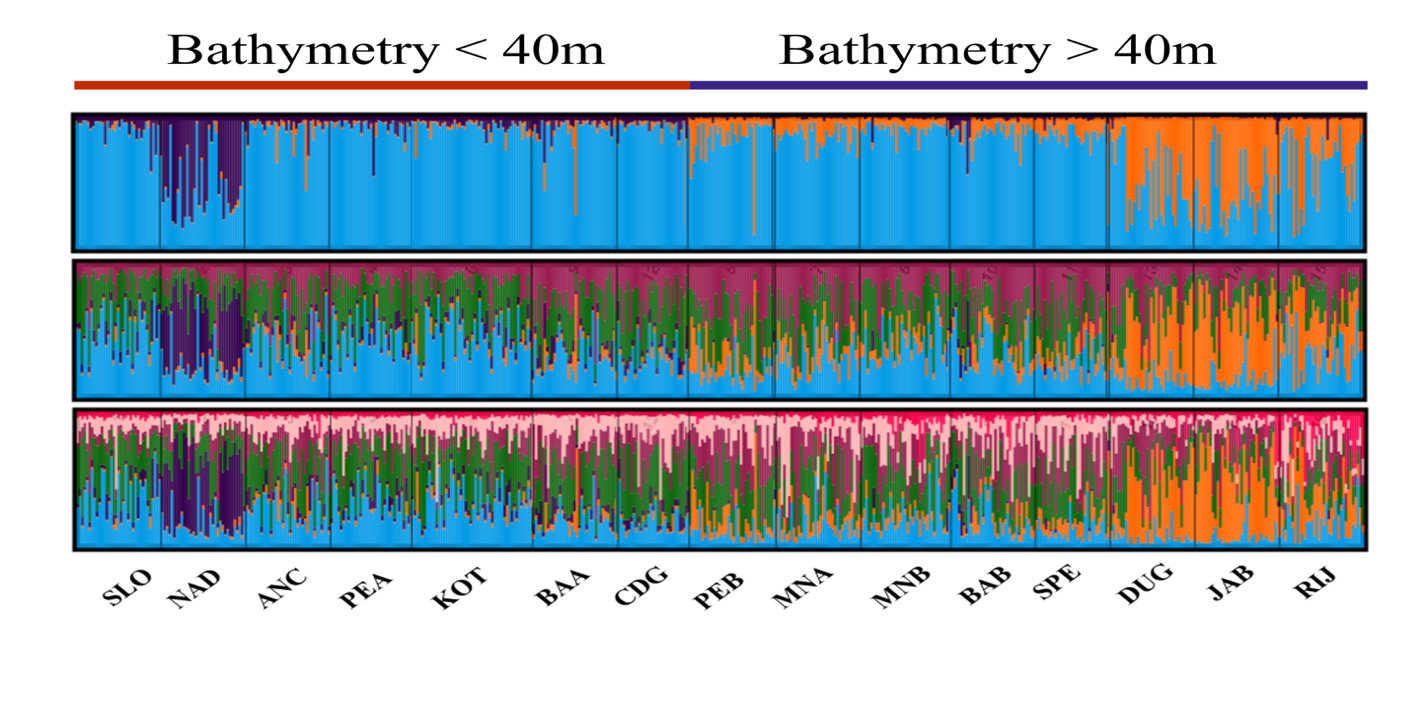

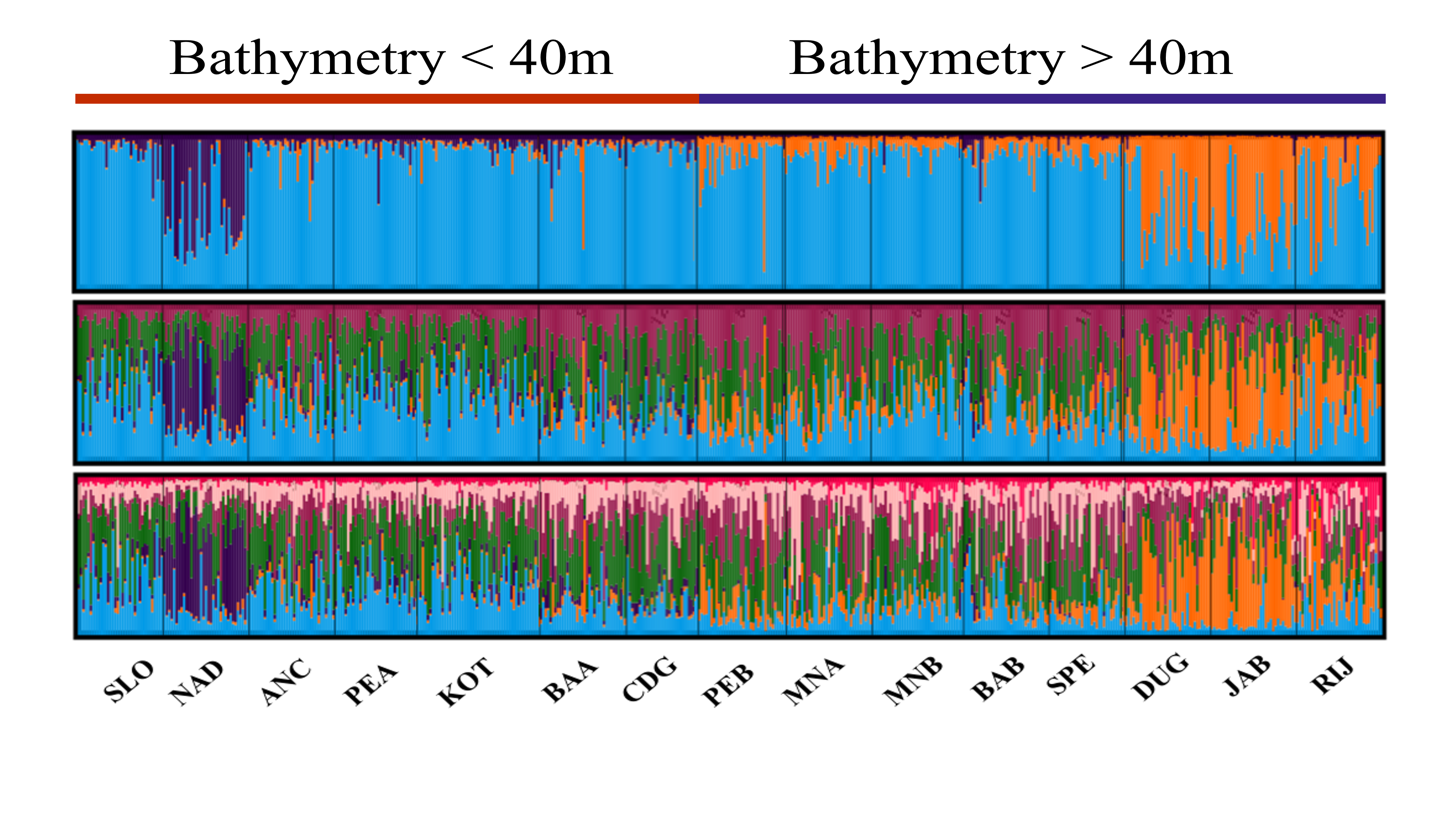

Supplement: S1 Fig — The simulations were run using i) all 13 loci, ii) only neutral loci and iii) only candidate outliers with and without the assistance of prior information (S1A–S1F Fig). Each simulation shows the mean logarithmic probability with standard deviation (SD) obtained for each K tested (1 < K < 10; figure on the left) and the delta K probability obtained after the use of Evanno method [48] (2 < K < 9; figure on the right). Additionally, a simulation was carried out dividing sampling sites based on bathymetry using a threshold of 50 m to detect population structure associate with potential occurrence of different anchovy species in the covered area (S1G Fig). (DOCX) [file pone.0153061.s001.docx]
