# Supplementary material for: Biocomplexity in Populations of European Anchovy in the Adriatic Sea
Source: PLoS One. 2016 Apr 13;11(4):e0153061. doi: 10.1371/journal.pone.0153061 (PMC4830579; doi:10.1371/journal.pone.0153061)

**S2_ Fig. Graphical plot of fdist/Lositan simulation.** Blue dots represent interpolation between mean values of *F*_ST_ and *H*_E_ in each marker (microsatellite locus) analyzed. Blue dots falling within light grey area represent neutral markers whereas those within the yellow and the red areas represent outlier markers under balancing and directional selection. respectively.


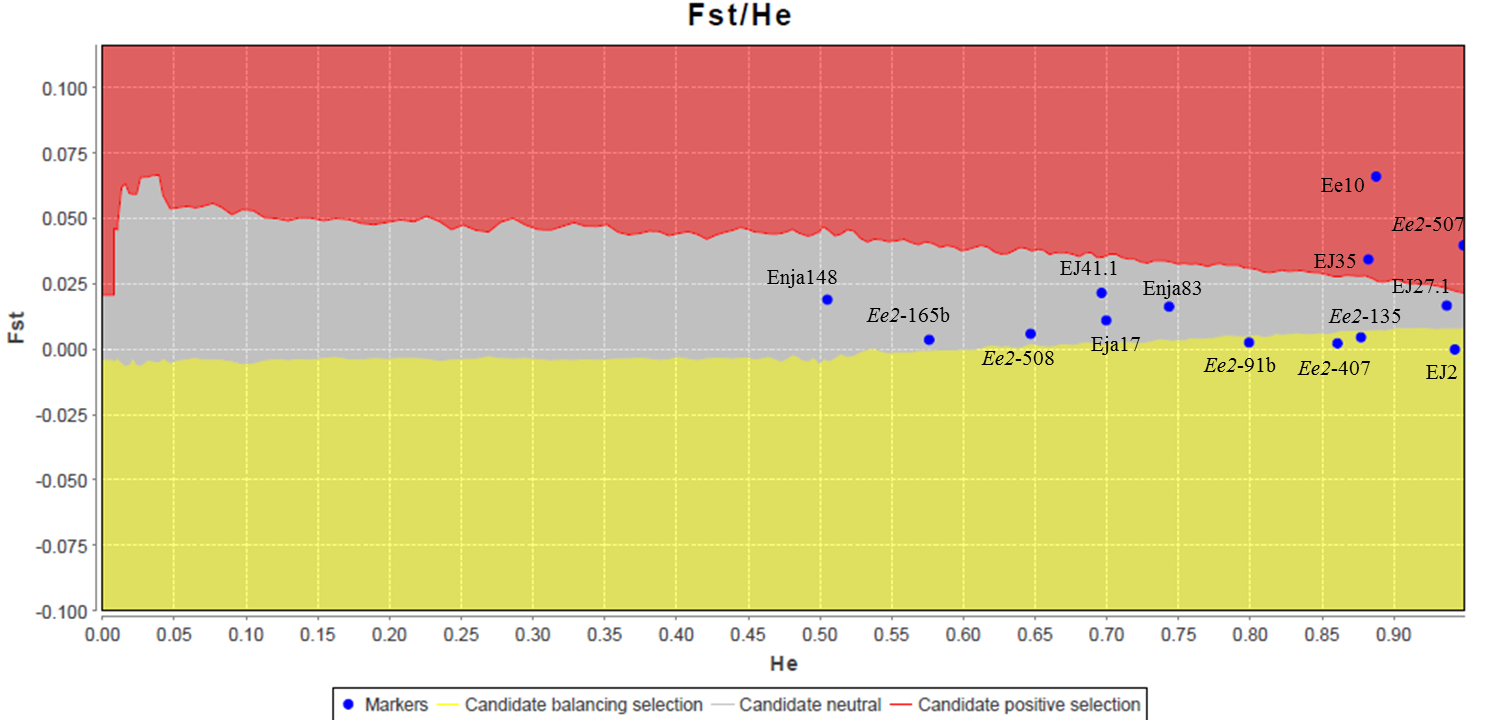

Supplement: S2 Fig — Blue dots represent interpolation between mean values of FST and HE in each marker (microsatellite locus) analyzed. Blue dots falling within light grey area represent neutral markers whereas those within the yellow and the red areas represent outlier markers under balancing and directional selection, respectively. (DOCX) [file pone.0153061.s002.docx]
