# Supplementary material for: Biocomplexity in Populations of European Anchovy in the Adriatic Sea
Source: PLoS One. 2016 Apr 13;11(4):e0153061. doi: 10.1371/journal.pone.0153061 (PMC4830579; doi:10.1371/journal.pone.0153061)

**S3_Fig. Graphical plot of Hierarchical Island Model simulations under the assumption of population structured in two (K = 2; S3_A Fig) and three (K = 3; S3_B Fig) clusters.** Dots represent the interpolation between mean values of *F*_ST_ and *H*_E_ in each marker (microsatellite locus) analyzed. Red, green and purple lines represent 1%, 5% and 95% percentiles of *F*_ST_ null distribution, respectively.

A)


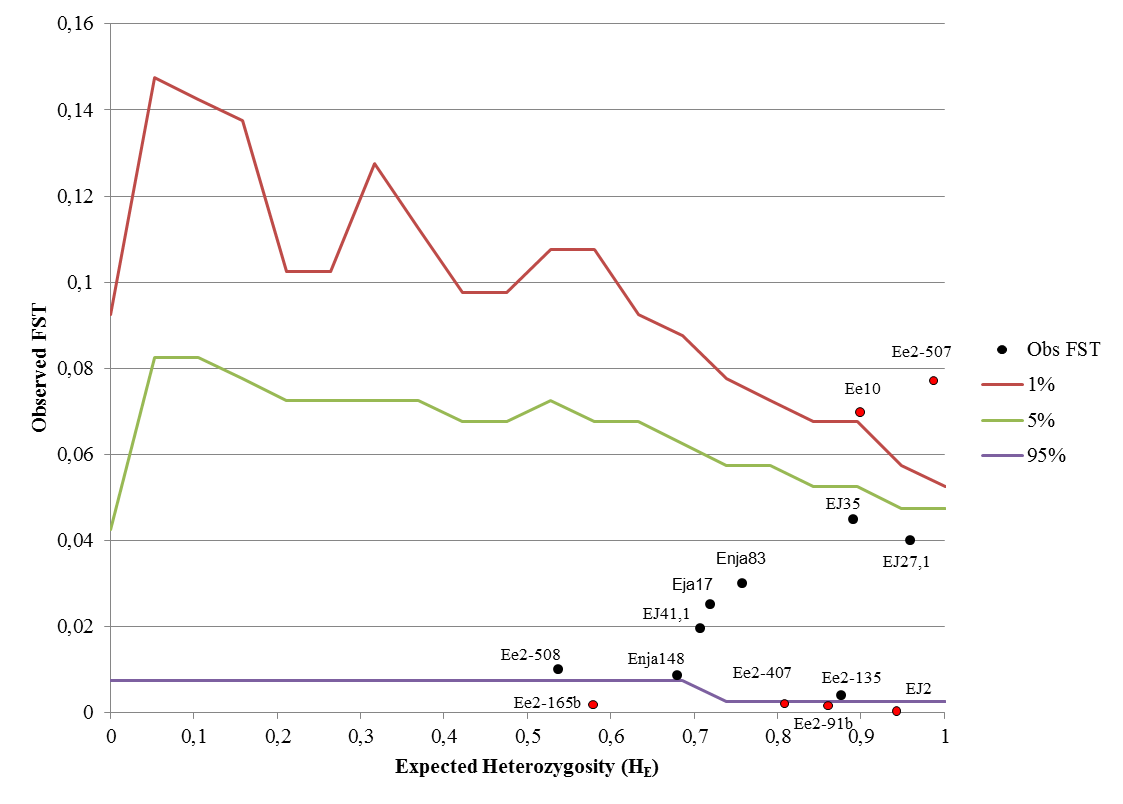


B)


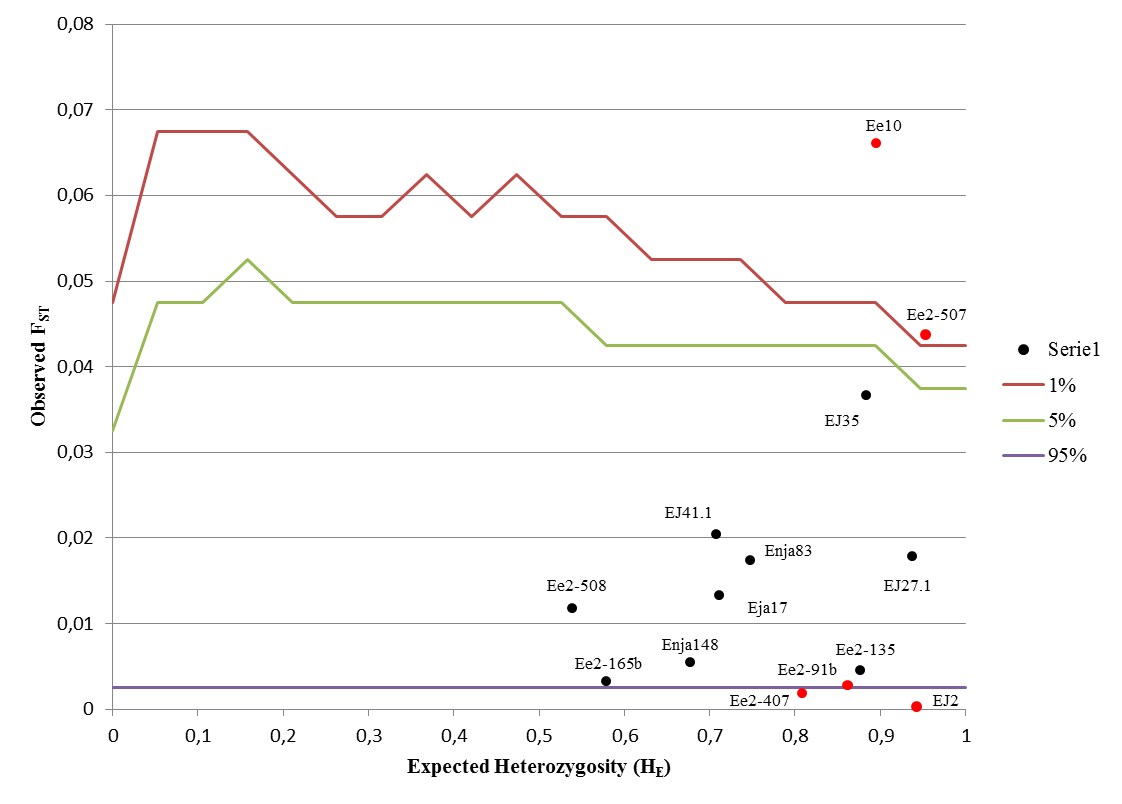

Supplement: S3 Fig — Dots represent the interpolation between mean values of FST and HE in each marker (microsatellite locus) analyzed. Red, green and purple lines represent 1%, 5% and 95% percentiles of FST null distribution, respectively. (DOCX) [file pone.0153061.s003.docx]
