# Supplementary material for: Biocomplexity in Populations of European Anchovy in the Adriatic Sea
Source: PLoS One. 2016 Apr 13;11(4):e0153061. doi: 10.1371/journal.pone.0153061 (PMC4830579; doi:10.1371/journal.pone.0153061)
Supplement: S1 File — (DOCX) [file pone.0153061.s004.docx]

**S1 File**

**Supporting Materials to Ruggeri et al., …..**

**Microsatellite PCR protocols**

PCR conditions were optimized for the 14 microsatellite loci using a touchdown amplification profile. The PCR profile included an initial denaturation step at 92°C for 5 min, followed by 10 cycles of 92°C/30 s denaturation, 40 s annealing (see Table S1 for starting annealing temperature) and 72°C/50 s extension. The starting annealing temperature was reduced of 0.5°C/cycle. Additional 25 cycles were performed with annealing temperature fixed at 5°C less than the starting annealing temperature. A step of final elongation at 72°C for 7 min was performed.

Three multiplex PCR mixes were optimized as follows:

- the multiplex A contained loci Ee2-91b, Ee2-407, EJ41.1 and Ee10 and was performed in a total volume of 20 μL.

- the multiplex B1 contained loci EJ27.1, EJ35, Enja83 and Ee2-507 and was performed in a total volume of 20 μL.

- the multiplex B2 contained loci EJ2, Ee2-135 and Eja17 and was performed in a total volume of 15 μL.

The PCR reactions for non-labelled loci (Enja148, Ee2-508 and Ee2-165b) were performed individually in a total volume of 10 μL, each.

PCR reaction mixture contained approximately 1-5 ng·μL^-1^ of genomic DNA, 0.025 U·μL^-1^ of Taq DNA polymerase (MyTaq DNA Polymerase, Bioline), 0.25 μM of each primer, 1× MyTaq Reaction Buffer (5mM dNTPs, 15 mM MgCl_2_, stabilizers and enhancers). A blank control was included in each PCR plate.

**Microsatellite Genotyping Procedures**

Labelled loci were analyzed by means of an Automated sequencer ABI-PRISM 3130xl Genetic Analyzer (Applied Biosystems) using LIZ500 as internal standard. The genotypes were obtained using the software PeakScanner^TM^ v.1.0 (freely available from Applied Biosystems, <http://www.appliedbiosystems.com/peakscanner.html>). Over 10% of the global dataset were re-genotyped to assure consistency of results. Raw genotyped data were refined using the binning procedure implemented in Flexibin (Amos et al., 2007). Non-labelled loci were genotyped using a 5% Polyacrylamide (Acrylamide-Bis-Acrylamide 19:1) denaturing sequencing gel and visualized by the silver staining protocol proposed by Bassam et al. (1991). The allele sizes were defined by comparison with the reaction sequence of pGEM-3Zf(+) (Promega sequencing kit). Reproducibility of the results were verified rerunning on each gel a subset of randomly chosen individuals that represented about 10% of total samples. In addition, genotyping error rates for non-labelled loci were limited by applying the allele ladder method as proposed by LaHood et al. (2002). The allele ladder is a pool of PCR products from multiple individuals and is representative of all or many of the alleles encountered in a given species for a particular locus (Moran et al. 2006). This method reduces the risks of misaligning alleles and consequently increases the accuracy of genotyping.

**Microsatellite data Power Analysis**

A simulation method implemented in an extended version of POWSIM 4.1 software (Ryman and Palm 2006) was used to assess the statistical power of the dataset, for detection of population differentiation. Tests were carried out testing the 15 localities using default parameters (1000 dememorizations, 100 batch and 1000 iterations per batch) and using several combinations of population divergence time (*t*) with an effective population size (*N*e) of 1000 individuals (*N*e/*t*: 1000/2, 1000/5; 1000/10, 1000/20.1, 1000/40.4; 1000/50.6, 1000/102.6). Each *F*_ST_ tested were simulated with 1000 replicates. The choice of testing for a fixed *N*_e_ of 1000 individuals was related to the detection of effective population size in European anchovy that are set in the low thousands of individuals (Ruggeri et al, in press). Different set of markers were comaperd: 1) all loci (13 microsatellite loci), ii) nine neutral loci and iii) 4 candidate outliers. The tests were comparatively carried out applying both Fisher's exact test and Chi squared test Here below the Table with results:

|  | **Fisher's exact test** | | | | **Chi squared** | | |  |
| --- | --- | --- | --- | --- | --- | --- | --- | --- |
| **Fst** | **all loci** | **neutral loci** | **outliers** | **all loci** | | **neutral loci** | **outliers** | |
| **0.0005** | 0.4700 | 0.3100 | 0.2100 | 0.4300 | | 0.2100 | 0.2100 | |
| **0,0010** | 0.8600 | 0.6800 | 0.5600 | 0.7900 | | 0.5300 | 0.5500 | |
| **0,0025** | 1.0000 | 0.9900 | 1.0000 | 1.0000 | | 0.9800 | 0.9800 | |
| **0,0050** | 1.0000 | 1.0000 | 1.0000 | 1.0000 | | 1.0000 | 1.0000 | |
| **0,0100** | 1.0000 | 1.0000 | 1.0000 | 1.0000 | | 1.0000 | 1.0000 | |
| **0,0200** | 1.0000 | 1.0000 | 1.0000 | 1.0000 | | 1.0000 | 1.0000 | |
| **0,0250** | 1.0000 | 1.0000 | 1.0000 | 1.0000 | | 1.0000 | 1.0000 | |
| **0,0500** | 1.0000 | 1.0000 | 1.0000 | 1.0000 | | 1.0000 | 1.0000 | |

The table explains the frequencies for each *F*_ST_ value from 0.0005 to 0.0500. The usage of all loci demonstrated to be adequate to detect *F*_ST_ values as low as 0.0010 in 79% or 86% of the replicates. Using exclusively neutral markers the power decreased (as expected) for the same *F*_ST_ vales to 53% and 68% of the replicates. The use of exclusively candidate outliers (despite only 4 loci) allowed enough power to detect *F*_ST_ of at least 0.025.

**Bibliographical support to S1 File**

Amos, W., Hoffman, J.I., Frodsham, A., Zhang, L., Best, S., Hill, A.V.S. 2007. Automated binning of microsatellite alleles: problems and solutions. Mol. Ecol. Notes 7: 10-14. doi: 10.1111/j.1471-8286.2006.01560.x

Bassam, B.J., Caetano-Anollés, G., Gresshoff, P.M. 1991. Fast and sensitive silver staining of DNA in polyacrylamide gels. Analyt. Biochem. 196(1): 80-83. doi:10.1016/0003-2697(91)90120-I.

LaHood, E.S., Moran, P., Olsen, J., Grant, W.S., and Park, L.K. 2002. Microsatellite allele ladders in two species of Pacific salmon: preparation and field-test results. Mol. Ecol. Notes, 2(2): 187–190. doi:10.1046/j.1471-8286.2002.00174.x.

Moran, P., Teel, D.J., LaHood, E.S., Drake, J., and Kalinowski, S. 2006. Standardizing multi-laboratory microsatellite data in Pacific salmon: an historical view of the future. Ecol. Fresh. Fish, 15(4): 597–605. doi:10.1111/j.1600-0633.2006.00201.x.

Ryman, N., Palm, S. 2006. POWSIM: a computer program for assessing statistical power when testing for genetic differentiation. *Mol. Ecol. Notes* 6: 600-602.

Ruggeri P., Splendiani, A., Di Muri, C., Fioravanti, T., Santojanni, A., Leonori, I., De Felice, A., Biagiotti, I., Carpi, P., Arneri, A., Nisi Cerioni, P., Giovannotti, M., Caputo Barucchi, V. In press. Coupling demographic and genetic variability from archived collections of European anchovy (*Engraulis encrasicolus*). Plos One.
