# Supplementary material for: Biocomplexity in Populations of European Anchovy in the Adriatic Sea
Source: PLoS One. 2016 Apr 13;11(4):e0153061. doi: 10.1371/journal.pone.0153061 (PMC4830579; doi:10.1371/journal.pone.0153061)
Supplement: S1 Table — (DOCX) [file pone.0153061.s005.docx]

**S1 Table.** Summary of the microsatellite loci and primers for their PCR amplification used in the present study.

| **Locus Name** | **Primers Sequences** | **Repeat motifs** | **T_A_** | **Fluor.** | **Multiplex** | **Species** | ***Authors*** |
| --- | --- | --- | --- | --- | --- | --- | --- |
| Ee2-91b | F: GGTCTTGAGCTTGGCATAGG | (CCGCA)_7_ | 60 | 6-FAM | A | *E. encrasicolus* | *28* |
|  | R: CCGGAAGACACTCTGCACAC |  |  |  |  |  |  |
| Ee2-407 | F: AGGAATCTCCTTCCCGTCTC | (CA)_13_ | 60 | VIC | A | *E. encrasicolus* | *28* |
|  | R: GTGGGTCTGTGGGTGTTTTG |  |  |  |  |  |  |
| Ej41-1 | F: TCTACCCCTGGAGGACACAC | (CACAA)_8_ | 55 | NED | A | *E. japonicus* | *26* |
|  | R: ACAGGGGGTTGAGAAAGAGG |  |  |  |  |  |  |
| Ee10 | F: GGTGGATGAAGTGGCAATCT | [(GT)_9_CT]_2_ | 54 | PET | A | *E. encrasicolus* | *27* |
|  | R: CTGGGGTGGCATAACTGAAG | [(GT)_2_CT]_3_ |  |  |  |  |  |
| Ej27.1 | F: GACTGTGAAGGAACGCTGGT | (GA)_36_ | 58 | 6-FAM | B1 | *E. japonicus* | *26* |
|  | R: AATAGGATTAGTCATCACAGGG |  |  |  |  |  |  |
| Ej35 | F: AGTGAGAGGACTCGCAAAGC | (TG)_15_ | 60 | PET | B1 | *E. japonicus* | *26* |
|  | R: CACACGAAGACAGACAAGCAA |  |  |  |  |  |  |
| Enja83 | F: AAGGGACATCGGGTAGTGA | (AC)_6_(TG)_7_ | 55 | NED | B1 | *E. japonicus* | *29* |
|  | R: AAGGCAAGTTCTCAGACGAG |  |  |  |  |  |  |
| Ee2-507 | F: GGAAGGGACCTAGATGGAGTG | (GAAA)_n_ | 60 | VIC | B1 | *E. encrasicolus* | *28* |
|  | R: ATCCCATTGATGTCCTGAGC |  |  |  |  |  |  |
| Eja17 | F: CCATTCAACTCCTCCCCAAGC | (CA)_7_ | 55 | 6-FAM | B2 | *E. japonicus* | *29* |
|  | R: GGCTCTTCAGCTCCCTGAGAC |  |  |  |  |  |  |
| Ej2 | F: AGCAAGGGAGCAAACAATC | (CT)_43_ | 58 | NED | B2 | *E. japonicus* | *26* |
|  | R: TGCAATTTGACAGAAACCACA |  |  |  |  |  |  |
| Ee2-135 | F:AGGGCAGTGACAGGAGAGTC | (ATTAG)_10_ | 55 | VIC | B2 | *E. encrasicolus* | *28* |
|  | R: TCGTTACCCTGCGTTTATACTG |  |  |  |  |  |  |
| Ee2-165b | F: GGGTGGGTTAAAGATGAAGC | (CCT)_7_ | 59 |  |  | *E. encrasicolus* | *28* |
|  | R: AGGGATCTTCAGGGAACCAG |  |  |  |  |  |  |
| Enja-148 | F: CTCCATCTTCCGTATTCT | (TG)_6_ | 55 |  |  | *E. japonicus* | *29* |
|  | R: GTAAATGATGTTGATGCTAA |  |  |  |  |  |  |
| Ee2-508 | F: CACATGCTCGCTAAACATTG | (AGG)_8_ | 55 |  |  | *E. encrasicolus* | *28* |
|  | R: ACCTGATGCTGCTTGGTAGC |  |  |  |  |  |  |
