# Supplementary material for: Biocomplexity in Populations of European Anchovy in the Adriatic Sea
Source: PLoS One. 2016 Apr 13;11(4):e0153061. doi: 10.1371/journal.pone.0153061 (PMC4830579; doi:10.1371/journal.pone.0153061)
Supplement: S2 Table — (DOCX) [file pone.0153061.s006.docx]

**S2 Table.** Values of the 13 environmental variables considered for each locality

| **Localities** | **T0** | **T10** | **T20** | **T-samp** | **S0** | **S10** | **S20** | **S-samp** | **Oxyg0** | **Oxyg10** | **Oxyg20** | **Oxyg-samp** | **Chla** |
| --- | --- | --- | --- | --- | --- | --- | --- | --- | --- | --- | --- | --- | --- |
| MNA | 24.02 | 20.99 | 17.02 | 20.99 | 37.99 | 38.43 | 38.53 | 38.43 | 4.77 | 5.23 | 5.58 | 5.23 | 0.199 |
| MNB | 23.92 | 21.04 | 17.15 | 15.20 | 38.02 | 38.43 | 38.54 | 38.67 | 4.77 | 5.25 | 5.62 | 5.22 | 0.095 |
| SLO | 25.98 | 23.59 | 18.75 | 23.59 | 35.98 | 37.10 | 37.93 | 37.10 | 5.09 | 4.61 | 3.20 | 4.61 | 0.919 |
| EMA | 24.68 | 22.99 | 18.38 | 15.03 | 34.83 | 36.46 | 37.82 | 37.98 | 4.93 | 5.09 | 5.33 | 4.46 | 0.436 |
| BAA | 21.47 | 19.94 | 17.51 | 17.51 | 37.82 | 37.92 | 38.07 | 38.07 | 5.24 | 5.39 | 5.57 | 5.39 | 0.358 |
| BAB | 21.96 | 20.52 | 18.26 | 13.65 | 37.80 | 37.97 | 38.16 | 38.46 | 5.20 | 5.39 | 5.57 | 5.32 | 0.153 |
| KOT | 24.50 | 23.03 | 17.83 | 23.03 | 38.43 | 38.48 | 38.59 | 38.48 | 4.76 | 5.11 | 5.68 | 5.11 | 0.01 |
| ANC | 24.30 | 23.26 | 18.23 | 15.24 | 35.31 | 36.51 | 37.86 | 38.05 | 4.98 | 5.06 | 5.32 | 5.71 | 0.565 |
| EMB | 23.85 | 23.08 | 19.36 | 14.22 | 37.55 | 37.73 | 38.17 | 38.37 | 4.99 | 5.11 | 5.64 | 5.97 | 0.087 |
| EMC | 21.13 | 20.83 | 18.99 | 13.00 | 38.58 | 38.30 | 38.17 | 37.87 | 5.10 | 5.29 | 5.56 | 5.30 | 0.135 |
| EMD | 23.04 | 22.29 | 20.97 | 18.04 | 39.07 | 38.52 | 38.21 | 38.26 | 4.97 | 5.16 | 5.56 | 5.84 | 0.205 |
| PEA | 25.33 | 25.21 | 22.02 | 25.21 | 36.82 | 37.24 | 37.85 | 37.24 | 4.84 | 4.88 | 5.46 | 4.88 | 0.441 |
| PEB | 25.26 | 25.06 | 22.13 | 13.91 | 36.46 | 36.94 | 37.70 | 38.34 | 4.87 | 4.89 | 5.38 | 6.14 | 0.287 |
| SPE | 20.17 | 19.33 | 17.23 | 15.99 | 37.41 | 37.75 | 37.97 | 38.12 | 5.94 | 6.12 | 6.37 | 6.06 | 0.691 |
| CDG | 15.37 | 15.27 | 15.09 | 15.27 | 37.52 | 37.53 | 37.57 | 37.61 | 5.81 | 5.80 | 5.81 | 5.78 | 0.074 |

Table S2. T0 = surface temperature (°C); T10 = 10 meters depth temperature (°C); T20 = 20 meters depth temperature (°C); T-samp = sampling depth temperature (°C); S0 = surface salinity (psu); S10 = 10 meters depth salinity (psu); S20 = 20 meters depth salinity (psu); S-samp = sampling depth salinity (psu); Oxyg0 = surface oxygen content (ml/l); Oxyg10 = 10 meters depth oxygen content (ml/l); Oxyg20 = 20 meters depth oxygen content (ml/l); Oxyg-samp = sampling depth oxygen content (ml/l); Chla = Chlorophyll-a surface concentration (mg/m^3^).
