# Supplementary material for: Biocomplexity in Populations of European Anchovy in the Adriatic Sea
Source: PLoS One. 2016 Apr 13;11(4):e0153061. doi: 10.1371/journal.pone.0153061 (PMC4830579; doi:10.1371/journal.pone.0153061)
Supplement: S3 Table — (DOCX) [file pone.0153061.s007.docx]

**S3 Table.** Summary of genetic variability observed at 14 microsatellite loci and a mtDNA genome portion in the sampled populations.

|  |  | ***MNA*** | ***MNB*** | ***SLO*** | ***NAD*** | | ***BAA*** | | ***BAB*** | | ***KOT*** | | ***ANC*** | | ***DUG*** | | ***JAB*** | | ***RIJ*** | | ***PEA*** | | ***PEB*** | | ***SPE*** | | ***CDG*** | |  | ***N*_A TOT_** | |  |
| --- | --- | --- | --- | --- | --- | --- | --- | --- | --- | --- | --- | --- | --- | --- | --- | --- | --- | --- | --- | --- | --- | --- | --- | --- | --- | --- | --- | --- | --- | --- | --- | --- |
| ***Ee2-91b*** | ***N*_A_** | 8 | 9 | 9 | | 10 | | 7 | | 10 | | 11 | | 10 | | 8 | | 9 | | 7 | | 8 | | 10 | | 8 | | 6 | |  | 15 | |
|  | ***N*** | 35 | 37 | 34 | | 29 | | 26 | | 32 | | 50 | | 30 | | 35 | | 35 | | 27 | | 35 | | 35 | | 23 | | 29 | |  |  | |
|  | ***f_null_*** | -0.029 | 0.041 | -0.015 | | 0.053 | | 0.057 | | 0.049 | | -0.064 | | 0.037 | | -0.061 | | 0.030 | | 0.070 | | 0.057 | | -0.015 | | 0.068 | | -0.062 | |  |  | |
|  | ***H_E_*** | 0.765 | 0.758 | 0.808 | | 0.835 | | 0.810 | | 0.821 | | 0.831 | | 0.814 | | 0.788 | | 0.808 | | 0.806 | | 0.800 | | 0.843 | | 0.839 | | 0.767 | |  |  | |
|  | ***H_O_*** | 0.800 | 0.676 | 0.823 | | 0.724 | | 0.692 | | 0.719 | | 0.940 | | 0.733 | | 0.886 | | 0.743 | | 0.667 | | 0.686 | | 0.857 | | 0.696 | | 0.862 | |  |  | |
|  | ***F*_IS_** | -0.046 | 0.109 | -0.019 | | 0.135 | | 0.148 | | 0.126 | | -0.132 | | 0.101 | | -0.126 | | 0.081 | | 0.176 | | 0.144 | | -0.017 | | 0.174 | | -0.126 | |  |  | |
|  | ***R*_S_** | 6.84 | 6.56 | 7.58 | | 9.06 | | 6.71 | | 8.99 | | 8.72 | | 8.59 | | 7.03 | | 7.67 | | 6.89 | | 7.06 | | 8.89 | | 7.63 | | 5.77 | |  | 7.60 | |
| ***Ee2-407*** | ***N*_A_** | 18 | 17 | 18 | | 17 | | 17 | | 19 | | 16 | | 16 | | 17 | | 13 | | 13 | | 20 | | 13 | | 17 | | 16 | |  | 38 | |
|  | ***N*** | 35 | 37 | 35 | | 34 | | 35 | | 34 | | 50 | | 35 | | 35 | | 35 | | 35 | | 35 | | 35 | | 30 | | 28 | |  |  | |
|  | ***f_null_*** | 0.053 | -0.013 | 0.011 | | **0.062** | | -0.020 | | **0.059** | | 0.013 | | 0.049 | | 0.006 | | 0.048 | | -0.036 | | -0.001 | | -0.029 | | 0.063 | | **0.075** | |  |  | |
|  | ***H_E_*** | 0.884 | 0.826 | 0.833 | | 0.895 | | 0.861 | | 0.889 | | 0.853 | | 0.845 | | 0.881 | | 0.843 | | 0.803 | | 0.896 | | 0.789 | | 0.901 | | 0.907 | |  |  | |
|  | ***H_O_*** | 0.771 | 0.838 | 0.800 | | 0.765 | | 0.886 | | 0.765 | | 0.820 | | 0.743 | | 0.857 | | 0.743 | | 0.857 | | 0.886 | | 0.829 | | 0.767 | | 0.750 | |  |  | |
|  | ***F*_IS_** | 0.129 | -0.014 | 0.040 | | 0.148 | | -0.030 | | 0.141 | | 0.039 | | 0.122 | | 0.028 | | 0.120 | | -0.068 | | 0.012 | | -0.051 | | 0.151 | | 0.176 | |  |  | |
|  | ***R*_S_** | 13.57 | 11.88 | 13.61 | | 13.66 | | 12.65 | | 14.81 | | 11.54 | | 12.10 | | 12.56 | | 10.81 | | 9.64 | | 14.80 | | 9.74 | | 13.66 | | 13.45 | |  | 12.57 | |
| ***EJ41.1*** | ***N*_A_** | 14 | 12 | 15 | | 14 | | 11 | | 10 | | 12 | | 12 | | 8 | | 10 | | 13 | | 11 | | 10 | | 9 | | 7 | |  | 25 | |
|  | ***N*** | 35 | 31 | 34 | | 35 | | 35 | | 34 | | 50 | | 35 | | 30 | | 28 | | 31 | | 35 | | 33 | | 30 | | 29 | |  |  | |
|  | ***f_null_*** | -0.002 | 0.031 | 0.032 | | 0.037 | | 0.072 | | 0.046 | | -0.019 | | 0.079 | | 0.099 | | **0.095** | | 0.061 | | -0.042 | | 0.029 | | -0.026 | | -0.041 | |  |  | |
|  | ***H_E_*** | 0.723 | 0.845 | 0.773 | | 0.611 | | 0.672 | | 0.613 | | 0.694 | | 0.717 | | 0.596 | | 0.670 | | 0.764 | | 0.710 | | 0.697 | | 0.669 | | 0.601 | |  |  | |
|  | ***H_O_*** | 0.714 | 0.774 | 0.706 | | 0.543 | | 0.543 | | 0.529 | | 0.720 | | 0.571 | | 0.467 | | 0.500 | | 0.645 | | 0.771 | | 0.636 | | 0.700 | | 0.655 | |  |  | |
|  | ***F*_IS_** | 0.013 | 0.085 | 0.088 | | 0.113 | | 0.194 | | 0.138 | | -0.037 | | 0.205 | | 0.219 | | 0.257 | | 0.158 | | -0.088 | | 0.088 | | -0.047 | | -0.092 | |  |  | |
|  | ***R*_S_** | 9.71 | 10.16 | 11.48 | | 10.14 | | 8.24 | | 7.33 | | 8.21 | | 9.05 | | 6.62 | | 8.61 | | 10.28 | | 8.59 | | 8.22 | | 7.38 | | 5.51 | |  | 8.64 | |
| ***Ee10*** | ***N*_A_** | 19 | 18 | 18 | | 17 | | 19 | | 18 | | 25 | | 15 | | 19 | | 18 | | 15 | | 21 | | 21 | | 16 | | 19 | |  | 37 | |
|  | ***N*** | 35 | 35 | 35 | | 32 | | 35 | | 33 | | 50 | | 35 | | 34 | | 31 | | 28 | | 35 | | 35 | | 30 | | 27 | |  |  | |
|  | ***f_null_*** | 0.042 | 0.060 | -0.024 | | 0.048 | | 0.032 | | **0.063** | | -0.040 | | 0.051 | | -0.021 | | 0.056 | | **0.114** | | 0.014 | | -0.064 | | -0.014 | | **0.065** | |  |  | |
|  | ***H_E_*** | 0.891 | 0.836 | 0.769 | | 0.852 | | 0.873 | | 0.889 | | 0.796 | | 0.880 | | 0.797 | | 0.755 | | 0.828 | | 0.808 | | 0.892 | | 0.821 | | 0.877 | |  |  | |
|  | ***H_O_*** | 0.800 | 0.714 | 0.800 | | 0.750 | | 0.800 | | 0.758 | | 0.860 | | 0.771 | | 0.824 | | 0.645 | | 0.607 | | 0.771 | | 1.000 | | 0.833 | | 0.741 | |  |  | |
|  | ***F*_IS_** | 0.104 | 0.147 | -0.042 | | 0.122 | | 0.084 | | 0.150 | | -0.081 | | 0.124 | | -0.034 | | 0.147 | | 0.270 | | 0.045 | | -0.123 | | -0.015 | | 0.158 | |  |  | |
|  | ***R*_S_** | 14.35 | 13.44 | 12.68 | | 12.87 | | 14.18 | | 13.58 | | 14.41 | | 12.24 | | 13.93 | | 13.57 | | 12.44 | | 14.55 | | 15.57 | | 12.91 | | 15.66 | |  | 13.76 | |
|  |  |  |  |  | |  | |  | |  | |  | |  | |  | |  | |  | |  | |  | |  | |  | |  |  | |
|  |  |  |  |  | |  | |  | |  | |  | |  | |  | |  | |  | |  | |  | |  | |  | |  |  | |
|  |  | ***MNA*** | ***MNB*** | ***SLO*** | | ***NAD*** | | ***BAA*** | | ***BAB*** | | ***KOT*** | | ***ANC*** | | ***DUG*** | | ***JAB*** | | ***RIJ*** | | ***PEA*** | | ***PEB*** | | ***SPE*** | | ***CDG*** | |  | ***N*_A TOT_** | |
| ***EJ27.1*** | ***N*_A_** | 23 | 27 | 26 | | 22 | | 22 | | 25 | | 25 | | 26 | | 17 | | 17 | | 21 | | 22 | | 25 | | 23 | | 23 | |  | 36 | |
|  | ***N*** | 35 | 37 | 35 | | 34 | | 35 | | 35 | | 49 | | 35 | | 35 | | 34 | | 34 | | 35 | | 35 | | 29 | | 28 | |  |  | |
|  | ***f_null_*** | 0.099 | 0.032 | 0.002 | | 0.010 | | -0.007 | | 0.042 | | **0.058** | | -0.022 | | -0.087 | | -0.054 | | -0.014 | | 0.064 | | 0.069 | | 0.095 | | 0.001 | |  |  | |
|  | ***H_E_*** | 0.949 | 0.940 | 0.960 | | 0.945 | | 0.914 | | 0.952 | | 0.939 | | 0.942 | | 0.852 | | 0.798 | | 0.841 | | 0.935 | | 0.947 | | 0.961 | | 0.948 | |  |  | |
|  | ***H_O_*** | 0.743 | 0.865 | 0.943 | | 0.912 | | 0.914 | | 0.857 | | 0.816 | | 0.971 | | 1.000 | | 0.882 | | 0.853 | | 0.800 | | 0.800 | | 0.759 | | 0.929 | |  |  | |
|  | ***F*_IS_** | **0.219** | 0.081 | 0.018 | | 0.036 | | 0.000 | | 0.101 | | 0.132 | | -0.031 | | -0.177 | | -0.107 | | -0.015 | | 0.147 | | 0.157 | | **0.213** | | 0.021 | |  |  | |
|  | ***R*_S_** | 18.97 | 19.53 | 20.55 | | 17.81 | | 16.63 | | 19.77 | | 18.66 | | 19.07 | | 13.39 | | 12.89 | | 15.86 | | 17.51 | | 19.05 | | 19.90 | | 19.27 | |  | 17.92 | |
| ***EJ35*** | ***N*_A_** | 15 | 13 | 14 | | 9 | | 14 | | 10 | | 13 | | 12 | | 9 | | 9 | | 10 | | 12 | | 16 | | 13 | | 14 | |  | 25 | |
|  | ***N*** | 35 | 37 | 35 | | 35 | | 35 | | 34 | | 50 | | 35 | | 35 | | 35 | | 35 | | 35 | | 35 | | 30 | | 29 | |  |  | |
|  | ***f_null_*** | 0.013 | -0.002 | -0.005 | | -0.102 | | 0.065 | | **0.064** | | 0.014 | | 0.011 | | -0.104 | | -0.126 | | -0.005 | | 0.034 | | -0.023 | | 0.043 | | 0.014 | |  |  | |
|  | ***H_E_*** | 0.836 | 0.901 | 0.831 | | 0.774 | | 0.907 | | 0.866 | | 0.833 | | 0.890 | | 0.797 | | 0.762 | | 0.832 | | 0.876 | | 0.913 | | 0.861 | | 0.904 | |  |  | |
|  | ***H_O_*** | 0.800 | 0.892 | 0.829 | | 0.943 | | 0.771 | | 0.735 | | 0.800 | | 0.857 | | 0.971 | | 0.971 | | 0.829 | | 0.800 | | 0.943 | | 0.767 | | 0.862 | |  |  | |
|  | ***F*_IS_** | 0.044 | 0.010 | 0.004 | | -0.222 | | 0.152 | | 0.153 | | 0.040 | | 0.038 | | -0.223 | | -0.280 | | 0.004 | | 0.088 | | -0.033 | | 0.111 | | 0.048 | |  |  | |
|  | ***R*_S_** | 11.42 | 11.60 | 10.43 | | 7.99 | | 12.38 | | 8.84 | | 9.75 | | 10.37 | | 7.64 | | 7.72 | | 8.74 | | 10.09 | | 13.44 | | 10.89 | | 12.79 | |  | 10.27 | |
| ***Enja83*** | ***N*_A_** | 9 | 8 | 13 | | 7 | | 7 | | 8 | | 14 | | 10 | | 6 | | 7 | | 8 | | 7 | | 10 | | 8 | | 9 | |  | 23 | |
|  | ***N*** | 31 | 36 | 35 | | 35 | | 35 | | 35 | | 48 | | 31 | | 35 | | 34 | | 35 | | 35 | | 35 | | 30 | | 27 | |  |  | |
|  | ***f_null_*** | **0.083** | -0.038 | -0.064 | | -0.085 | | -0.021 | | 0.058 | | -0.010 | | -0.006 | | -0.119 | | -0.047 | | -0.093 | | -0.014 | | 0.020 | | -0.080 | | -0.015 | |  |  | |
|  | ***H_E_*** | 0.771 | 0.722 | 0.784 | | 0.616 | | 0.746 | | 0.739 | | 0.803 | | 0.776 | | 0.695 | | 0.639 | | 0.683 | | 0.757 | | 0.761 | | 0.741 | | 0.766 | |  |  | |
|  | ***H_O_*** | 0.613 | 0.778 | 0.886 | | 0.743 | | 0.771 | | 0.629 | | 0.813 | | 0.774 | | 0.886 | | 0.706 | | 0.829 | | 0.771 | | 0.714 | | 0.867 | | 0.778 | |  |  | |
|  | ***F*_IS_** | 0.207 | -0.078 | -0.132 | | -0.210 | | -0.035 | | 0.151 | | -0.012 | | 0.002 | | -0.280 | | -0.106 | | -0.217 | | -0.019 | | 0.062 | | -0.173 | | -0.016 | |  |  | |
|  | ***R*_S_** | 7.93 | 6.60 | 9.74 | | 5.57 | | 6.44 | | 6.87 | | 9.55 | | 8.54 | | 5.43 | | 5.73 | | 6.57 | | 6.32 | | 8.28 | | 7.08 | | 7.73 | |  | 7.23 | |
| ***Ee2-507*** | ***N*_A_** | 24 | 22 | 19 | | 14 | | 22 | | 22 | | 29 | | 24 | | 20 | | 17 | | 16 | | 26 | | 21 | | 22 | | 25 | |  | 41 | |
|  | ***N*** | 35 | 37 | 35 | | 35 | | 35 | | 35 | | 50 | | 34 | | 35 | | 35 | | 34 | | 35 | | 35 | | 30 | | 29 | |  |  | |
|  | ***f_null_*** | 0.001 | 0.027 | 0.031 | | -0.179 | | -0.015 | | -0.003 | | -0.009 | | -0.003 | | -0.051 | | -0.123 | | -0.089 | | 0.003 | | 0.012 | | 0.053 | | 0.041 | |  |  | |
|  | ***H_E_*** | 0.958 | 0.957 | 0.930 | | 0.707 | | 0.957 | | 0.951 | | 0.953 | | 0.949 | | 0.862 | | 0.793 | | 0.821 | | 0.963 | | 0.952 | | 0.952 | | 0.959 | |  |  | |
|  | ***H_O_*** | 0.943 | 0.892 | 0.857 | | 1.000 | | 0.971 | | 0.943 | | 0.960 | | 0.941 | | 0.943 | | 1.000 | | 0.971 | | 0.943 | | 0.914 | | 0.833 | | 0.862 | |  |  | |
|  | ***F*_IS_** | 0.016 | 0.069 | 0.080 | | **-0.423** | | -0.015 | | 0.009 | | -0.008 | | 0.008 | | -0.096 | | -0.266 | | -0.186 | | 0.021 | | 0.040 | | 0.127 | | 0.103 | |  |  | |
|  | ***R*_S_** | 19.61 | 18.60 | 15.27 | | 9.52 | | 18.66 | | 18.23 | | 19.63 | | 19.00 | | 14.62 | | 11.99 | | 12.27 | | 20.59 | | 18.04 | | 18.18 | | 20.66 | |  | 17.00 | |
| ***Eja17*** | ***N*_A_** | 8 | 8 | 7 | | 7 | | 6 | | 6 | | 9 | | 7 | | 7 | | 7 | | 6 | | 9 | | 8 | | 5 | | 6 | |  | 15 | |
|  | ***N*** | 29 | 33 | 29 | | 35 | | 35 | | 35 | | 49 | | 29 | | 35 | | 35 | | 33 | | 30 | | 35 | | 22 | | 29 | |  |  | |
|  | ***f_null_*** | 0.078 | 0.046 | 0.035 | | 0.074 | | 0.006 | | 0.069 | | 0.025 | | 0.065 | | -0.027 | | 0.066 | | 0.035 | | 0.041 | | 0.055 | | 0.040 | | 0.047 | |  |  | |
|  | ***H_E_*** | 0.733 | 0.790 | 0.765 | | 0.765 | | 0.706 | | 0.698 | | 0.702 | | 0.747 | | 0.595 | | 0.600 | | 0.610 | | 0.750 | | 0.764 | | 0.672 | | 0.676 | |  |  | |
|  | ***H_O_*** | 0.586 | 0.697 | 0.690 | | 0.543 | | 0.686 | | 0.571 | | 0.653 | | 0.621 | | 0.629 | | 0.486 | | 0.546 | | 0.667 | | 0.657 | | 0.591 | | 0.586 | |  |  | |
|  | ***F*_IS_** | 0.203 | 0.119 | 0.100 | | 0.198 | | 0.029 | | 0.184 | | 0.070 | | 0.171 | | -0.057 | | 0.192 | | 0.108 | | 0.112 | | 0.141 | | 0.124 | | 0.135 | |  |  | |
|  | ***R*_S_** | 6.85 | 6.97 | 6.49 | | 5.68 | | 5.67 | | 5.05 | | 6.76 | | 6.73 | | 5.42 | | 6.00 | | 4.97 | | 7.39 | | 7.19 | | 4.73 | | 5.53 | |  | 6.10 | |
|  |  |  |  |  | |  | |  | |  | |  | |  | |  | |  | |  | |  | |  | |  | |  | |  |  | |
|  |  | ***MNA*** | ***MNB*** | ***SLO*** | | ***NAD*** | | ***BAA*** | | ***BAB*** | | ***KOT*** | | ***ANC*** | | ***DUG*** | | ***JAB*** | | ***RIJ*** | | ***PEA*** | | ***PEB*** | | ***SPE*** | | ***CDG*** | |  | ***N*_A TOT_** | |
| ***EJ2*** | ***N*_A_** | 21 | 20 | 15 | | 22 | | 21 | | 21 | | 25 | | 22 | | 22 | | 18 | | 19 | | 21 | | 24 | | 20 | | 23 | |  | 32 | |
|  | ***N*** | 35 | 37 | 35 | | 34 | | 35 | | 35 | | 50 | | 35 | | 34 | | 35 | | 35 | | 35 | | 35 | | 30 | | 29 | |  |  | |
|  | ***f_null_*** | -0.007 | 0.018 | 0.068 | | 0.012 | | 0.025 | | -0.007 | | 0.014 | | 0.025 | | 0.076 | | 0.049 | | 0.096 | | 0.007 | | 0.015 | | -0.020 | | 0.038 | |  |  | |
|  | ***H_E_*** | 0.944 | 0.939 | 0.913 | | 0.950 | | 0.947 | | 0.942 | | 0.936 | | 0.947 | | 0.955 | | 0.936 | | 0.940 | | 0.941 | | 0.957 | | 0.944 | | 0.951 | |  |  | |
|  | ***H_O_*** | 0.943 | 0.892 | 0.771 | | 0.912 | | 0.886 | | 0.943 | | 0.900 | | 0.886 | | 0.794 | | 0.829 | | 0.743 | | 0.914 | | 0.914 | | 0.967 | | 0.862 | |  |  | |
|  | ***F*_IS_** | 0.001 | 0.050 | 0.157 | | 0.040 | | 0.066 | | -0.001 | | 0.038 | | 0.065 | | 0.170 | | 0.116 | | **0.212** | | 0.029 | | 0.045 | | -0.024 | | 0.095 | |  |  | |
|  | ***R*_S_** | 17.36 | 17.00 | 13.07 | | 17.89 | | 17.60 | | 16.78 | | 17.60 | | 17.81 | | 18.29 | | 15.90 | | 16.01 | | 17.03 | | 19.26 | | 16.95 | | 19.15 | |  | 17.18 | |
| ***Ee2-135*** | ***N*_A_** | 12 | 14 | 13 | | 10 | | 10 | | 12 | | 13 | | 11 | | 11 | | 10 | | 10 | | 12 | | 10 | | 12 | | 12 | |  | 16 | |
|  | ***N*** | 35 | 37 | 35 | | 35 | | 35 | | 35 | | 50 | | 35 | | 35 | | 35 | | 34 | | 35 | | 35 | | 30 | | 29 | |  |  | |
|  | ***f_null_*** | -0.046 | 0.036 | -0.015 | | 0.038 | | -0.001 | | -0.069 | | -0.029 | | -0.043 | | -0.002 | | -0.024 | | -0.014 | | -0.074 | | -0.006 | | -0.080 | | -0.063 | |  |  | |
|  | ***H_E_*** | 0.897 | 0.891 | 0.899 | | 0.854 | | 0.869 | | 0.884 | | 0.874 | | 0.875 | | 0.865 | | 0.854 | | 0.870 | | 0.875 | | 0.858 | | 0.866 | | 0.865 | |  |  | |
|  | ***H_O_*** | 0.971 | 0.811 | 0.914 | | 0.771 | | 0.857 | | 1.000 | | 0.920 | | 0.943 | | 0.857 | | 0.886 | | 0.882 | | 1.000 | | 0.857 | | 1.000 | | 0.966 | |  |  | |
|  | ***F*_IS_** | -0.084 | 0.091 | -0.018 | | 0.098 | | 0.014 | | -0.133 | | -0.053 | | -0.079 | | 0.009 | | -0.038 | | -0.014 | | -0.145 | | 0.001 | | -0.158 | | -0.199 | |  |  | |
|  | ***R*_S_** | 10.73 | 11.39 | 11.38 | | 8.94 | | 9.31 | | 10.06 | | 10.63 | | 9.91 | | 9.41 | | 9.00 | | 9.24 | | 10.11 | | 8.75 | | 10.42 | | 10.43 | |  | 9.98 | |
| ***Ee2-508*** | ***N*_A_** | 6 | 7 | 10 | | 6 | | 6 | | 5 | | 8 | | 6 | | 6 | | 8 | | 7 | | 8 | | 6 | | 4 | | 7 | |  | 11 | |
|  | ***N*** | 27 | 31 | 24 | | 27 | | 23 | | 27 | | 38 | | 19 | | 34 | | 35 | | 34 | | 30 | | 25 | | 21 | | 29 | |  |  | |
|  | ***f_null_*** | 0.069 | 0.079 | **0.127** | | **0.095** | | **0.118** | | 0.072 | | **0.077** | | **0.109** | | 0.078 | | 0.039 | | 0.052 | | 0.065 | | **0.102** | | 0.098 | | 0.057 | |  |  | |
|  | ***H_E_*** | 0.724 | 0.621 | 0.832 | | 0.732 | | 0.691 | | 0.648 | | 0.691 | | 0.671 | | 0.604 | | 0.645 | | 0.559 | | 0.723 | | 0.661 | | 0.652 | | 0.620 | |  |  | |
|  | ***H_O_*** | 0.593 | 0.484 | 0.583 | | 0.556 | | 0.478 | | 0.519 | | 0.553 | | 0.474 | | 0.471 | | 0.571 | | 0.471 | | 0.600 | | 0.480 | | 0.476 | | 0.517 | |  |  | |
|  | ***F*_IS_** | 0.184 | 0.223 | 0.303 | | 0.245 | | 0.313 | | 0.203 | | 0.202 | | 0.300 | | 0.223 | | 0.116 | | 0.161 | | 0.173 | | 0.278 | | 0.274 | | 0.168 | |  |  | |
|  | ***R*_S_** | 5.62 | 6.12 | 9.53 | | 5.97 | | 5.80 | | 4.89 | | 6.83 | | 6.00 | | 5.25 | | 6.21 | | 5.45 | | 7.11 | | 5.69 | | 4.00 | | 5.62 | |  | 6.00 | |
| ***Ee2-165b*** | ***N*_A_** | 5 | 5 | 7 | | 4 | | 4 | | 6 | | 5 | | 5 | | 8 | | 5 | | 4 | | 4 | | 6 | | 6 | | 5 | |  | 12 | |
|  | ***N*** | 35 | 37 | 35 | | 33 | | 35 | | 35 | | 49 | | 35 | | 34 | | 32 | | 35 | | 35 | | 35 | | 30 | | 29 | |  |  | |
|  | ***f_null_*** | 0.002 | 0.080 | -0.032 | | -0.015 | | -0.027 | | 0.042 | | -0.029 | | 0.051 | | 0.079 | | -0.042 | | -0.013 | | -0.026 | | 0.028 | | -0.059 | | 0.023 | |  |  | |
|  | ***H_E_*** | 0.525 | 0.654 | 0.642 | | 0.591 | | 0.565 | | 0.649 | | 0.592 | | 0.579 | | 0.638 | | 0.630 | | 0.559 | | 0.511 | | 0.595 | | 0.423 | | 0.456 | |  |  | |
|  | ***H_O_*** | 0.514 | 0.514 | 0.686 | | 0.606 | | 0.600 | | 0.571 | | 0.633 | | 0.486 | | 0.500 | | 0.688 | | 0.571 | | 0.543 | | 0.543 | | 0.500 | | 0.414 | |  |  | |
|  | ***F*_IS_** | 0.020 | 0.217 | -0.069 | | -0.026 | | -0.063 | | 0.121 | | -0.069 | | 0.163 | | 0.219 | | -0.093 | | -0.022 | | -0.063 | | 0.089 | | -0.185 | | 0.093 | |  |  | |
|  | ***R*_S_** | 4.35 | 4.96 | 5.57 | | 3.97 | | 3.90 | | 5.44 | | 4.31 | | 4.53 | | 6.29 | | 4.58 | | 3.89 | | 3.45 | | 5.04 | | 5.24 | | 4.54 | |  | 4.67 | |
| ***Enja-148*** | ***N*_A_** | 6 | 5 | 6 | | 5 | | 4 | | 6 | | 7 | | 4 | | 7 | | 5 | | 5 | | 4 | | 4 | | 6 | | 5 | |  | 16 | |
|  | ***N*** | 28 | 36 | 23 | | 32 | | 23 | | 35 | | 34 | | 19 | | 21 | | 27 | | 23 | | 35 | | 25 | | 22 | | 18 | |  |  | |
|  | ***f_null_*** | **0.122** | 0.075 | **0.151** | | 0.005 | | **0.173** | | 0.018 | | **0.140** | | **0.168** | | **0.176** | | **0.104** | | **0.134** | | **0.097** | | **0.127** | | **0.122** | | **0.176** | |  |  | |
|  | ***H_E_*** | 0.563 | 0.383 | 0.617 | | 0.468 | | 0.487 | | 0.482 | | 0.490 | | 0.548 | | 0.656 | | 0.460 | | 0.587 | | 0.491 | | 0.682 | | 0.571 | | 0.532 | |  |  | |
|  | ***H_O_*** | 0.393 | 0.278 | 0.391 | | 0.438 | | 0.261 | | 0.486 | | 0.294 | | 0.316 | | 0.400 | | 0.321 | | 0.391 | | 0.343 | | 0.480 | | 0.409 | | 0.278 | |  |  | |
|  | ***F*_IS_** | 0.306 | 0.278 | 0.371 | | 0.067 | | 0.470 | | -0.009 | | 0.403 | | 0.430 | | 0.397 | | 0.360 | | 0.339 | | 0.306 | | 0.300 | | 0.288 | | 0.485 | |  |  | |
|  | ***R*_S_** | 5.51 | 3.50 | 5.69 | | 4.30 | | 4.00 | | 4.54 | | 5.27 | | 3.95 | | 6.78 | | 4.27 | | 4.78 | | 3.41 | | 7.35 | | 5.78 | | 5.00 | |  | 5.45 | |
|  |  |  |  |  | |  | |  | |  | |  | |  | |  | |  | |  | |  | |  | |  | |  | |  |  | |
|  |  | ***MNA*** | ***MNB*** | ***SLO*** | | ***NAD*** | | ***BAA*** | | ***BAB*** | | ***KOT*** | | ***ANC*** | | ***DUG*** | | ***JAB*** | | ***RIJ*** | | ***PEA*** | | ***PEB*** | | ***SPE*** | | ***CDG*** | |  | ***N*_A TOT_** | |
| ***Average**** | ***N*_AM_** | 14.0 | 13.9 | 14.2 | | 12.2 | | 12.8 | | 13.2 | | 15.8 | | 13.5 | | 12.2 | | 11.4 | | 11.5 | | 13.9 | | 13.9 | | 12.5 | | 13.2 | |  |  | |
|  | ***H_E_*** | 0.815 | 0.821 | 0.826 | | 0.722 | | 0.809 | | 0.811 | | 0.808 | | 0.818 | | 0.763 | | 0.749 | | 0.763 | | 0.811 | | 0.818 | | 0.792 | | 0.792 | |  |  | |
|  | ***H_O_*** | 0.753 | 0.756 | 0.791 | | 0.751 | | 0.758 | | 0.734 | | 0.799 | | 0.752 | | 0.776 | | 0.742 | | 0.728 | | 0.781 | | 0.780 | | 0.750 | | 0.753 | |  |  | |
|  | ***F*_IS_** | **0.077** | **0.081** | 0.043 | | 0.028 | | 0.064 | | **0.096** | | 0.011 | | **0.082** | | -0.016 | | 0.009 | | 0.046 | | 0.038 | | 0.046 | | 0.054 | | 0.051 | |  |  | |
|  | ***R*_S_** | 9.8 | 9.7 | 9.8 | | 8.6 | | 9.2 | | 9.4 | | 9.8 | | 8.4 | | 8.1 | | 8.2 | | 9.6 | | 9.8 | | 9.3 | | 10.7 | | 11.2 | |  |  | |
|  |  |  |  |  | |  | |  | |  | |  | |  | |  | |  | |  | |  | |  | |  | |  | |  |  | |

S3 Table. *N*_A_ _TOT_ = Number of alleles observed in a specific locus; *N*_A_ = number of alleles observed per location; *N* = number of individuals correctly genotyped; *N*_AM_ = mean number of alleles observed per location; *f_null_ =* null allele frequency; *H*_O_ = observed heterozygosity; *H*_E_ = expected heterozygosity; *F*_IS_ = inbreeding coefficient estimates; *R*_S_ = allelic richness estimates standardized at 19 individuals. Bold *F*_IS_ values are significant (<0.05) after a sequential Bonferroni correction [37]. Bold *f_null_* values are those still showed null allele signals after applying Brookfield null allele correction method [32]; * The average was produced on the basis of all microsatellite loci excluding Enja-148. For the local sample codes, see Table 1-A.
