# Supplementary material for: Biocomplexity in Populations of European Anchovy in the Adriatic Sea
Source: PLoS One. 2016 Apr 13;11(4):e0153061. doi: 10.1371/journal.pone.0153061 (PMC4830579; doi:10.1371/journal.pone.0153061)
Supplement: S4 Table — In S4A Table are showed values from G'ST and GST while in S4B Table the values from DEST. (DOCX) [file pone.0153061.s008.docx]

S4 Table A. Hedrick`s G`_ST_ [41] values are shown below the diagonal, while Nei`s G_ST_ [40] values above the diagonal. Significant values (P<0.0005) are shown in bold.

|  | MNA | MNB | SLO | NAD | BAA | BAB | KOT | ANC | DUG | JAB | RIJ | PEA | PEB | SPE | CDG |
| --- | --- | --- | --- | --- | --- | --- | --- | --- | --- | --- | --- | --- | --- | --- | --- |
| MNA | / | 0.008 | **0.015** | **0.031** | -0.001 | 0.003 | 0.009 | 0.004 | **0.017** | **0.025** | **0.017** | 0.008 | 0.002 | 0.003 | -0.001 |
| MNB | 0.004 | / | 0.009 | **0.036** | **0.013** | 0.010 | 0.006 | 0.004 | **0.035** | **0.040** | **0.015** | 0.005 | **0.015** | **0.021** | **0.017** |
| SLO | **0.008** | 0.004 | / | **0.037** | **0.016** | 0.009 | 0.005 | **0.014** | **0.042** | **0.048** | **0.020** | 0.005 | **0.020** | **0.029** | **0.030** |
| NAD | **0.016** | **0.019** | **0.019** | / | **0.025** | **0.020** | **0.036** | **0.030** | **0.048** | **0.057** | **0.038** | **0.030** | **0.029** | **0.035** | **0.027** |
| BAA | -0.001 | **0.006** | **0.008** | **0.012** | / | 0.003 | **0.013** | 0.005 | 0.011 | **0.018** | **0.015** | **0.010** | -0.004 | 0.004 | -0.001 |
| BAB | 0.001 | 0.005 | 0.005 | **0.010** | 0.001 | / | 0.006 | 0.002 | **0.019** | **0.029** | **0.011** | 0.004 | 0.004 | 0.008 | 0.007 |
| KOT | 0.005 | 0.003 | 0.003 | **0.018** | **0.006** | 0.003 | / | 0.008 | **0.026** | **0.034** | 0.010 | 0.002 | **0.015** | **0.021** | **0.017** |
| ANC | 0.002 | 0.002 | **0.007** | **0.015** | 0.002 | 0.001 | 0.004 | / | **0.018** | **0.027** | **0.014** | 0.007 | 0.004 | 0.011 | 0.005 |
| DUG | **0.009** | **0.018** | **0.021** | **0.025** | 0.006 | **0.010** | **0.013** | **0.009** | / | -0.003 | **0.014** | **0.032** | **0.015** | **0.021** | **0.019** |
| JAB | **0.013** | **0.021** | **0.025** | **0.030** | **0.009** | **0.015** | **0.017** | **0.014** | -0.002 | / | **0.016** | **0.039** | **0.022** | **0.027** | **0.028** |
| RIJ | **0.008** | **0.007** | **0.010** | **0.019** | **0.007** | **0.006** | 0.005 | **0.007** | **0.007** | **0.008** | / | **0.013** | **0.021** | **0.025** | **0.022** |
| PEA | 0.004 | 0.003 | 0.003 | **0.015** | **0.005** | 0.002 | 0.001 | 0.003 | **0.016** | **0.020** | **0.007** | / | **0.017** | **0.016** | **0.013** |
| PEB | 0.001 | **0.007** | **0.010** | **0.014** | -0.002 | 0.002 | **0.008** | 0.002 | **0.007** | **0.011** | **0.011** | **0.008** | / | 0.007 | 0.006 |
| SPE | 0.002 | **0.011** | **0.014** | **0.018** | 0.002 | 0.004 | **0.010** | 0.005 | **0.011** | **0.014** | **0.013** | **0.008** | 0.004 | / | -0.001 |
| CDG | 0.000 | **0.008** | **0.015** | **0.013** | 0.000 | 0.003 | **0.008** | 0.002 | **0.010** | **0.014** | **0.011** | **0.006** | 0.003 | 0.000 | / |

S4 Table B. Jost`s D [42], D_EST_ values. Significant values (P<0.0005) are shown in bold.

|  | MNA | MNB | SLO | NAD | BAA | BAB | KOT | ANC | DUG | JAB | RIJ | PEA | PEB | SPE | CDG |
| --- | --- | --- | --- | --- | --- | --- | --- | --- | --- | --- | --- | --- | --- | --- | --- |
| MNA | / |  |  |  |  |  |  |  |  |  |  |  |  |  |  |
| MNB | 0.038 | / |  |  |  |  |  |  |  |  |  |  |  |  |  |
| SLO | **0.070** | 0.042 | / |  |  |  |  |  |  |  |  |  |  |  |  |
| NAD | **0.122** | **0.149** | **0.151** | / |  |  |  |  |  |  |  |  |  |  |  |
| BAA | -0.006 | **0.057** | **0.072** | **0.095** | / |  |  |  |  |  |  |  |  |  |  |
| BAB | 0.013 | **0.046** | 0.041 | **0.078** | 0.011 | / |  |  |  |  |  |  |  |  |  |
| KOT | 0.039 | 0.027 | 0.024 | **0.139** | **0.054** | 0.024 | / |  |  |  |  |  |  |  |  |
| ANC | 0.018 | 0.020 | **0.065** | **0.120** | 0.020 | 0.008 | 0.033 | / |  |  |  |  |  |  |  |
| DUG | **0.066** | **0.139** | **0.169** | **0.166** | 0.042 | **0.072** | **0.098** | **0.069** | / |  |  |  |  |  |  |
| JAB | **0.093** | **0.154** | **0.187** | **0.194** | **0.066** | **0.107** | **0.123** | **0.099** | -0.009 | / |  |  |  |  |  |
| RIJ | **0.063** | **0.056** | **0.079** | **0.130** | **0.054** | **0.042** | 0.035 | **0.053** | **0.047** | **0.049** | / |  |  |  |  |
| PEA | 0.033 | 0.023 | 0.024 | **0.118** | **0.043** | 0.018 | 0.007 | 0.030 | **0.123** | **0.145** | **0.049** | / |  |  |  |
| PEB | 0.011 | **0.069** | **0.095** | **0.114** | -0.019 | 0.016 | **0.068** | 0.017 | **0.057** | **0.080** | **0.081** | **0.074** | / |  |  |
| SPE | 0.013 | **0.091** | **0.125** | **0.132** | 0.016 | 0.032 | **0.084** | 0.045 | **0.077** | **0.093** | **0.092** | **0.065** | 0.031 | / |  |
| CDG | -0.004 | **0.070** | **0.131** | **0.098** | -0.004 | 0.027 | **0.069** | 0.021 | **0.069** | **0.097** | **0.078** | **0.052** | 0.025 | -0.003 | / |
