# Supplementary material for: Biocomplexity in Populations of European Anchovy in the Adriatic Sea
Source: PLoS One. 2016 Apr 13;11(4):e0153061. doi: 10.1371/journal.pone.0153061 (PMC4830579; doi:10.1371/journal.pone.0153061)
Supplement: S5 Table — The numbers within the table refer to the individuals that were allocated outside the sampling localities examined in this study (receiving sampling locations). The original sampling sites detected for each migrant are reported along the first row (native sampling locations). The S5B Table provide values for the demographic estimator Theta (Θ), while S5C Table shows migration rates (M) of historical gene flow. The sampling location in the first line represent the source locations while the sampling location in the first column the receiving locations. (DOCX) [file pone.0153061.s009.docx]

S5_A Table

|  |  | **NATIVE SAMPLING LOCATIONS** | | | | | | | | | | | | | | |
| --- | --- | --- | --- | --- | --- | --- | --- | --- | --- | --- | --- | --- | --- | --- | --- | --- |
|  |  | ***MNA*** | ***MNB*** | ***SLO*** | ***NAD*** | ***BAA*** | ***BAB*** | ***KOT*** | ***ANC*** | ***DUG*** | ***JAB*** | ***RIJ*** | ***PEA*** | ***PEB*** | ***SPE*** | ***CDG*** |
| **RECEIVING SAMPLING LOCATIONS** | ***MNA*** |  |  |  |  |  |  |  |  |  |  |  | 1 |  |  |  |
|  | ***MNB*** |  |  |  |  |  |  |  | 1 |  |  |  |  |  |  |  |
|  | ***SLO*** |  | 1 |  |  |  |  |  | 1 |  |  |  |  | 1 |  |  |
|  | ***NAD*** | 1 | 1 |  |  |  |  |  | 2 |  | 1 |  | 1 |  |  |  |
|  | ***BAA*** |  |  |  |  |  |  |  |  |  |  |  |  |  | 1 |  |
|  | ***BAB*** | 2 |  |  |  |  |  | 1 |  | 1 |  |  |  |  | 1 |  |
|  | ***KOT*** |  |  |  |  | 1 |  |  |  |  |  | 1 |  |  |  |  |
|  | ***ANC*** |  |  |  |  |  |  |  |  |  |  | 1 | 1 |  |  |  |
|  | ***DUG*** | 1 | 1 |  |  | 1 |  |  | 1 |  |  |  |  |  |  |  |
|  | ***JAB*** | 3 |  |  |  |  |  |  |  |  |  |  |  |  |  |  |
|  | ***RIJ*** | 2 |  |  |  |  |  | 1 |  | 1 |  |  |  |  |  |  |
|  | ***PEA*** |  | 1 |  |  |  |  |  | 1 |  |  | 1 |  | 1 |  |  |
|  | ***PEB*** | 1 |  |  |  |  |  |  |  |  |  |  |  |  |  |  |
|  | ***SPE*** |  |  |  |  | 1 | 1 |  |  |  |  |  |  |  |  |  |
|  | ***CDG*** | 1 |  |  |  | 1 |  |  |  |  |  |  |  |  |  |  |

S5_B Table

| THETA (𝚯) | MEAN VALUE | CI 2.5% | CI 97.5% |
| --- | --- | --- | --- |
| **ANC** | 0.015 | 0.007 | 0.018 |
| **BAA** | 0.010 | 0.000 | 0.013 |
| **BAB** | 0.039 | 0.029 | 0.046 |
| **CDG** | 0.003 | 0.001 | 0.005 |
| **NAD** | 0.023 | 0.013 | 0.022 |
| **DUG** | 0.014 | 0.009 | 0.017 |
| **JAB** | 0.046 | 0.029 | 0.045 |
| **RIJ** | 0.026 | 0.019 | 0.030 |
| **KOT** | 0.009 | 0.000 | 0.012 |
| **MNA** | 0.005 | 0.003 | 0.007 |
| **MNB** | 0.028 | 0.036 | 0.052 |
| **PEA** | 0.008 | 0.004 | 0.012 |
| **PEB** | 0.031 | 0.000 | 0.005 |
| **SLO** | 0.012 | 0.006 | 0.014 |
| **SPE** | 0.023 | 0.002 | 0.012 |

S5_C Table

| **M VALUES** | **ANC** | **BAA** | **BAB** | **CDG** | **NAD** | **DUG** | **JAB** | **RIJ** | **KOT** | **MNA** | **MNB** | **PEA** | **PEB** | **SLO** | **SPE** |
| --- | --- | --- | --- | --- | --- | --- | --- | --- | --- | --- | --- | --- | --- | --- | --- |
| **ANC** | / | 163.06 | 170.35 | 46.19 | 697.85 | 464.88 | 58.11 | 168.56 | 146.28 | 566.37 | 239.54 | **667.99** | 170.57 | 25.77 | 478.64 |
| **BAA** | 155.30 | / | 61.19 | 777.57 | 156.79 | 361.36 | 47.07 | 139.09 | 502.98 | 233.53 | 227.70 | 238.82 | 216.61 | 247.11 | 332.05 |
| **BAB** | 362.36 | **646.64** | / | 516.40 | 40.57 | 211.32 | 120.00 | 187.39 | 48.76 | 230.92 | 627.63 | 119.96 | 102.56 | 308.10 | 318.94 |
| **CDG** | **735.09** | 19.15 | **567.70** | / | 142.35 | 727.25 | 90.30 | 252.11 | 87.50 | 199.23 | 100.11 | 360.42 | 270.02 | 261.80 | **578.03** |
| **NAD** | 76.90 | 263.34 | 66.87 | 122.71 | / | 136.52 | 18.50 | 138.27 | **736.04** | 79.93 | 70.51 | 323.79 | 145.04 | **538.47** | 556.55 |
| **DUG** | 395.07 | 177.90 | 317.18 | 452.48 | 190.96 | / | 149.17 | 64.59 | 54.97 | 379.27 | 104.01 | 116.00 | 78.69 | 323.77 | 106.66 |
| **JAB** | 49.91 | 310.02 | 277.80 | 556.76 | 151.06 | 499.03 | / | 101.87 | 156.45 | 74.80 | 151.51 | 648.48 | 93.63 | 154.08 | 103.00 |
| **RIJ** | 686.49 | 88.44 | 128.86 | 44.03 | 231.50 | 488.34 | 298.34 | / | 209.86 | **786.42** | 541.27 | 341.93 | 184.06 | 316.06 | 200.39 |
| **KOT** | 461.10 | 255.46 | 295.46 | 116.44 | 797.84 | 122.65 | 229.07 | 60.62 | / | 106.99 | 489.36 | 99.91 | 686.56 | 207.59 | 63.73 |
| **MNA** | 519.89 | 116.15 | 174.76 | **902.00** | 76.19 | 232.29 | 299.95 | 317.28 | 141.25 | / | 366.87 | 450.95 | 85.21 | 114.55 | 91.20 |
| **MNB** | 459.43 | 381.29 | 101.35 | 177.92 | 614.20 | 89.03 | 535.04 | **723.41** | 329.71 | 142.10 | / | 187.26 | 730.60 | 152.29 | 304.12 |
| **PEA** | 175.20 | 63.13 | 144.25 | 711.34 | 691.86 | **944.01** | 486.92 | 103.28 | 61.16 | 49.16 | 485.79 | / | 548.78 | 120.47 | 442.16 |
| **PEB** | 440.48 | 282.40 | 37.96 | 209.35 | **892.44** | 86.93 | **620.28** | 64.73 | 89.10 | 419.68 | 259.70 | 230.42 | / | 215.24 | 40.16 |
| **SLO** | 220.45 | 159.60 | 188.16 | 695.52 | 31.48 | 486.81 | 258.91 | 138.39 | 166.79 | 44.22 | 239.29 | 205.58 | 240.68 | / | 144.59 |
| **SPE** | 381.44 | 85.30 | 322.63 | 113.65 | 72.79 | 663.58 | 705.12 | 422.21 | 477.59 | 192.24 | **882.87** | 363.45 | **834.12** | 401.60 | / |
|  | **5119.11** | 3011.88 | 2854.50 | **5442.34** | 4787.87 | **5513.99** | 3916.77 | 2881.78 | 3208.45 | 3504.85 | 4786.15 | 4354.95 | 4387.14 | 3386.90 | 3760.22 |
|  | source |  |  | source |  | source |  |  |  |  |  |  |  |  |  |
