# Supplementary material for: Biocomplexity in Populations of European Anchovy in the Adriatic Sea
Source: PLoS One. 2016 Apr 13;11(4):e0153061. doi: 10.1371/journal.pone.0153061 (PMC4830579; doi:10.1371/journal.pone.0153061)
Supplement: S6 Table — Values in red are those exceeding the threshold of significance set at ±2.58. (DOCX) [file pone.0153061.s010.docx]

**S6_Table**

|  | **MNA *vs*** |  |  |  |  |  |  |  |  |  |  |  |  |  |
| --- | --- | --- | --- | --- | --- | --- | --- | --- | --- | --- | --- | --- | --- | --- |
| Locus | MNB | SLO | NAD | BAA | BAB | KOT | ANC | DUG | JAB | RIJ | PEA | PEB | SPE | CDG |
| Ee91b | 0.07 | -0.42 | -0.73 | -0.44 | -0.57 | -0.69 | -0.49 | -0.21 | -0.42 | -0.40 | -0.33 | -0.83 | -0.78 | -0.02 |
| Ee407 | 0.82 | 0.74 | -0.21 | 0.37 | -0.09 | 0.48 | 0.59 | 0.04 | 0.62 | 1.08 | -0.23 | 1.22 | -0.33 | -0.46 |
| EJ41,1 | -1.21 | -0.42 | 0.77 | 0.38 | 0.76 | 0.22 | 0.05 | 0.86 | 0.39 | -0.34 | 0.10 | 0.20 | 0.40 | 0.83 |
| Ee10 | 0.84 | 1.55 | 0.62 | 0.32 | 0.04 | 1.28 | 0.21 | 1.27 | 1.67 | 0.93 | 1.17 | -0.02 | 1.01 | 0.25 |
| EJ27,1 | 0.32 | -0.51 | 0.13 | 1.03 | -0.13 | 0.33 | 0.23 | 2.14 | 2.78 | 2.29 | 0.46 | 0.06 | -0.53 | 0.02 |
| EJ35 | -1.02 | 0.06 | 0.66 | -1.16 | -0.41 | 0.04 | -0.82 | 0.45 | 0.78 | 0.06 | -0.57 | -1.29 | -0.34 | -1.10 |
| Enja83 | 0.41 | -0.13 | 1.14 | 0.22 | 0.27 | -0.32 | -0.05 | 0.61 | 0.99 | 0.70 | 0.12 | 0.09 | 0.26 | 0.04 |
| Ee507 | 0.05 | 1.00 | 3.96 | 0.04 | 0.29 | 0.23 | 0.37 | 2.39 | 3.22 | 2.92 | -0.25 | 0.26 | 0.26 | -0.05 |
| Eja17 | -0.51 | -0.27 | 0.43 | 0.21 | 0.26 | 0.23 | -0.11 | 0.94 | 0.91 | 0.84 | -0.14 | -0.26 | 0.45 | 0.42 |
| EJ2 | 0.18 | 0.88 | -0.22 | -0.12 | 0.06 | 0.27 | -0.11 | -0.44 | 0.26 | 0.11 | 0.10 | -0.52 | -0.01 | -0.28 |
| Ee135 | 0.12 | -0.02 | 0.71 | 0.49 | 0.25 | 0.41 | 0.40 | 0.55 | 0.72 | 0.48 | 0.39 | 0.65 | 0.55 | 0.56 |
| Ee508m | 0.71 | -1.04 | -0.07 | 0.25 | 0.54 | 0.25 | 0.38 | 0.82 | 0.56 | 1.07 | 0.01 | 0.45 | 0.52 | 0.72 |
| Ee2-165b | -0.77 | -0.69 | -0.37 | -0.23 | -0.73 | -0.38 | -0.30 | -0.66 | -0.61 | -0.19 | 0.07 | -0.40 | 0.54 | 0.37 |

|  | **MNB *vs*** |  |  |  |  |  |  |  |  |  |  |  |  |
| --- | --- | --- | --- | --- | --- | --- | --- | --- | --- | --- | --- | --- | --- |
| Locus | SLO | NAD | BAA | BAB | KOT | ANC | DUG | JAB | RIJ | PEA | PEB | SPE | CDG |
| Ee91b | -0.49 | -0.80 | -0.51 | -0.63 | -0.76 | -0.56 | -0.28 | -0.48 | -0.47 | -0.40 | -0.90 | -0.85 | -0.09 |
| Ee407 | -0.08 | -1.03 | -0.45 | -0.90 | -0.34 | -0.23 | -0.77 | -0.20 | 0.26 | -1.05 | 0.41 | -1.15 | -1.27 |
| EJ41,1 | 0.79 | 1.98 | 1.59 | 1.97 | 1.43 | 1.27 | 2.07 | 1.60 | 0.87 | 1.32 | 1.42 | 1.61 | 2.04 |
| Ee10 | 0.71 | -0.22 | -0.52 | -0.80 | 0.44 | -0.63 | 0.43 | 0.84 | 0.09 | 0.33 | -0.86 | 0.17 | -0.59 |
| EJ27,1 | -0.84 | -0.19 | 0.71 | -0.46 | 0.01 | -0.10 | 1.82 | 2.45 | 1.96 | 0.14 | -0.26 | -0.86 | -0.30 |
| EJ35 | 1.08 | 1.69 | -0.14 | 0.61 | 1.06 | 0.20 | 1.47 | 1.80 | 1.08 | 0.45 | -0.27 | 0.68 | -0.08 |
| Enja83 | -0.53 | 0.73 | -0.19 | -0.14 | -0.73 | -0.46 | 0.21 | 0.58 | 0.29 | -0.29 | -0.32 | -0.15 | -0.37 |
| Ee507 | 0.95 | 3.91 | -0.01 | 0.24 | 0.18 | 0.32 | 2.34 | 3.17 | 2.87 | -0.30 | 0.21 | 0.21 | -0.10 |
| Eja17 | 0.24 | 0.94 | 0.72 | 0.77 | 0.74 | 0.40 | 1.44 | 1.42 | 1.35 | 0.37 | 0.25 | 0.96 | 0.93 |
| EJ2 | 0.71 | -0.39 | -0.30 | -0.12 | 0.10 | -0.28 | -0.62 | 0.09 | -0.06 | -0.07 | -0.69 | -0.19 | -0.45 |
| Ee135 | -0.14 | 0.59 | 0.37 | 0.13 | 0.29 | 0.28 | 0.44 | 0.60 | 0.36 | 0.27 | 0.53 | 0.43 | 0.44 |
| Ee508m | -1.75 | -0.78 | -0.46 | -0.17 | -0.46 | -0.33 | 0.10 | -0.15 | 0.36 | -0.70 | -0.26 | -0.19 | 0.00 |
| Ee2-165b | 0.08 | 0.39 | 0.54 | 0.03 | 0.39 | 0.46 | 0.11 | 0.16 | 0.57 | 0.84 | 0.37 | 1.30 | 1.13 |

|  | **SLO vs** |  |  |  |  |  |  |  |  |  |  |  |
| --- | --- | --- | --- | --- | --- | --- | --- | --- | --- | --- | --- | --- |
| Locus | NAD | BAA | BAB | KOT | ANC | DUG | JAB | RIJ | PEA | PEB | SPE | CDG |
| Ee91b | -0.31 | -0.02 | -0.14 | -0.27 | -0.07 | 0.21 | 0.01 | 0.02 | 0.09 | -0.41 | -0.36 | 0.41 |
| Ee407 | -0.95 | -0.37 | -0.83 | -0.26 | -0.15 | -0.70 | -0.13 | 0.33 | -0.97 | 0.48 | -1.07 | -1.20 |
| EJ41,1 | 1.19 | 0.80 | 1.18 | 0.64 | 0.47 | 1.28 | 0.81 | 0.08 | 0.53 | 0.63 | 0.82 | 1.25 |
| Ee10 | -0.93 | -1.23 | -1.51 | -0.27 | -1.35 | -0.28 | 0.12 | -0.62 | -0.39 | -1.57 | -0.54 | -1.30 |
| EJ27,1 | 0.65 | 1.54 | 0.38 | 0.85 | 0.74 | 2.65 | 3.29 | 2.80 | 0.97 | 0.58 | -0.02 | 0.54 |
| EJ35 | 0.61 | -1.21 | -0.47 | -0.02 | -0.87 | 0.39 | 0.72 | 0.00 | -0.63 | -1.34 | -0.39 | -1.15 |
| Enja83 | 1.26 | 0.34 | 0.40 | -0.19 | 0.08 | 0.74 | 1.12 | 0.82 | 0.24 | 0.21 | 0.38 | 0.17 |
| Ee507 | 2.96 | -0.96 | -0.71 | -0.77 | -0.63 | 1.39 | 2.22 | 1.92 | -1.25 | -0.74 | -0.74 | -1.05 |
| Eja17 | 0.70 | 0.48 | 0.54 | 0.51 | 0.16 | 1.21 | 1.18 | 1.12 | 0.13 | 0.01 | 0.72 | 0.69 |
| EJ2 | -1.10 | -1.00 | -0.82 | -0.61 | -0.99 | -1.32 | -0.62 | -0.77 | -0.78 | -1.40 | -0.90 | -1.16 |
| Ee135 | 0.74 | 0.52 | 0.27 | 0.44 | 0.42 | 0.58 | 0.74 | 0.50 | 0.42 | 0.68 | 0.57 | 0.59 |
| Ee508m | 0.97 | 1.29 | 1.58 | 1.29 | 1.42 | 1.85 | 1.60 | 2.11 | 1.04 | 1.49 | 1.55 | 1.75 |
| Ee2-165b | 0.31 | 0.46 | -0.05 | 0.31 | 0.38 | 0.03 | 0.08 | 0.50 | 0.76 | 0.29 | 1.22 | 1.05 |

|  | **NAD vs** |  |  |  |  |  |  |  |  |  |  |
| --- | --- | --- | --- | --- | --- | --- | --- | --- | --- | --- | --- |
| Locus | BAA | BAB | KOT | ANC | DUG | JAB | RIJ | PEA | PEB | SPE | CDG |
| Ee91b | 0.29 | 0.17 | 0.04 | 0.24 | 0.52 | 0.32 | 0.33 | 0.40 | -0.10 | -0.05 | 0.72 |
| Ee407 | 0.58 | 0.12 | 0.69 | 0.80 | 0.25 | 0.82 | 1.29 | -0.02 | 1.43 | -0.12 | -0.25 |
| EJ41,1 | -0.39 | -0.01 | -0.55 | -0.71 | 0.09 | -0.38 | -1.11 | -0.66 | -0.56 | -0.37 | 0.06 |
| Ee10 | -0.30 | -0.58 | 0.66 | -0.42 | 0.65 | 1.05 | 0.31 | 0.54 | -0.64 | 0.39 | -0.37 |
| EJ27,1 | 0.90 | -0.27 | 0.20 | 0.10 | 2.01 | 2.64 | 2.15 | 0.33 | -0.07 | -0.67 | -0.11 |
| EJ35 | -1.82 | -1.08 | -0.63 | -1.48 | -0.22 | 0.11 | -0.61 | -1.24 | -1.95 | -1.00 | -1.76 |
| Enja83 | -0.92 | -0.86 | -1.46 | -1.19 | -0.52 | -0.15 | -0.44 | -1.02 | -1.05 | -0.88 | -1.09 |
| Ee507 | -3.92 | -3.67 | -3.73 | -3.59 | -1.57 | -0.74 | -1.04 | -4.21 | -3.70 | -3.70 | -4.01 |
| Eja17 | -0.22 | -0.16 | -0.19 | -0.54 | 0.51 | 0.48 | 0.42 | -0.57 | -0.69 | 0.02 | -0.01 |
| EJ2 | 0.10 | 0.28 | 0.49 | 0.11 | -0.22 | 0.48 | 0.33 | 0.32 | -0.30 | 0.21 | -0.06 |
| Ee135 | -0.22 | -0.47 | -0.30 | -0.31 | -0.16 | 0.01 | -0.23 | -0.32 | -0.06 | -0.16 | -0.15 |
| Ee508m | 0.31 | 0.61 | 0.31 | 0.45 | 0.88 | 0.62 | 1.14 | 0.07 | 0.52 | 0.58 | 0.78 |
| Ee2-165b | 0.15 | -0.36 | -0.01 | 0.07 | -0.28 | -0.24 | 0.18 | 0.45 | -0.03 | 0.91 | 0.74 |

|  | **BAA vs** |  |  |  |  |  |  |  |  |  | **BAB vs** |  |  |  |
| --- | --- | --- | --- | --- | --- | --- | --- | --- | --- | --- | --- | --- | --- | --- |
| Locus | BAB | KOT | ANC | DUG | JAB | RIJ | PEA | PEB | SPE | CDG | KOT | ANC | DUG | JAB |
| Ee91b | -0.12 | -0.25 | -0.05 | 0.23 | 0.03 | 0.04 | 0.11 | -0.39 | -0.34 | 0.43 | -0.12 | 0.08 | 0.35 | 0.15 |
| Ee407 | -0.46 | 0.11 | 0.22 | -0.33 | 0.25 | 0.71 | -0.60 | 0.85 | -0.70 | -0.83 | 0.57 | 0.67 | 0.13 | 0.70 |
| EJ41,1 | 0.38 | -0.16 | -0.33 | 0.48 | 0.01 | -0.72 | -0.27 | -0.17 | 0.02 | 0.45 | -0.54 | -0.70 | 0.10 | -0.37 |
| Ee10 | -0.28 | 0.96 | -0.12 | 0.95 | 1.35 | 0.61 | 0.85 | -0.34 | 0.69 | -0.07 | 1.24 | 0.17 | 1.24 | 1.64 |
| EJ27,1 | -1.16 | -0.70 | -0.80 | 1.11 | 1.75 | 1.26 | -0.57 | -0.97 | -1.56 | -1.01 | 0.47 | 0.36 | 2.27 | 2.91 |
| EJ35 | 0.74 | 1.20 | 0.34 | 1.60 | 1.94 | 1.21 | 0.58 | -0.13 | 0.82 | 0.06 | 0.45 | -0.40 | 0.86 | 1.19 |
| Enja83 | 0.06 | -0.54 | -0.27 | 0.40 | 0.77 | 0.48 | -0.10 | -0.13 | 0.04 | -0.18 | -0.59 | -0.32 | 0.34 | 0.72 |
| Ee507 | 0.25 | 0.20 | 0.34 | 2.35 | 3.18 | 2.88 | -0.29 | 0.22 | 0.22 | -0.09 | -0.06 | 0.08 | 2.10 | 2.93 |
| Eja17 | 0.06 | 0.03 | -0.32 | 0.73 | 0.70 | 0.64 | -0.35 | -0.47 | 0.24 | 0.21 | -0.03 | -0.38 | 0.67 | 0.64 |
| EJ2 | 0.18 | 0.39 | 0.02 | -0.32 | 0.38 | 0.24 | 0.22 | -0.40 | 0.11 | -0.16 | 0.21 | -0.16 | -0.50 | 0.20 |
| Ee135 | -0.25 | -0.08 | -0.09 | 0.06 | 0.23 | -0.01 | -0.10 | 0.16 | 0.06 | 0.07 | 0.17 | 0.15 | 0.31 | 0.47 |
| Ee508m | 0.29 | 0.00 | 0.14 | 0.57 | 0.31 | 0.82 | -0.24 | 0.20 | 0.27 | 0.47 | -0.29 | -0.16 | 0.27 | 0.02 |
| Ee2-165b | -0.51 | -0.15 | -0.08 | -0.43 | -0.38 | 0.03 | 0.30 | -0.17 | 0.76 | 0.59 | 0.35 | 0.43 | 0.07 | 0.12 |

|  |  |  |  |  |  | **KOT vs** |  |  |  |  |  |  |  |
| --- | --- | --- | --- | --- | --- | --- | --- | --- | --- | --- | --- | --- | --- |
| Locus | RIJ | PEA | PEB | SPE | CDG | ANC | DUG | JAB | RIJ | PEA | PEB | SPE | CDG |
| Ee91b | 0.16 | 0.23 | -0.27 | -0.21 | 0.55 | 0.20 | 0.47 | 0.27 | 0.28 | 0.36 | -0.14 | -0.09 | 0.67 |
| Ee407 | 1.16 | -0.14 | 1.31 | -0.24 | -0.37 | 0.11 | -0.44 | 0.13 | 0.59 | -0.71 | 0.74 | -0.81 | -0.94 |
| EJ41,1 | -1.10 | -0.65 | -0.55 | -0.36 | 0.07 | -0.17 | 0.64 | 0.17 | -0.56 | -0.11 | -0.02 | 0.18 | 0.61 |
| Ee10 | 0.90 | 1.13 | -0.06 | 0.97 | 0.21 | -1.08 | -0.01 | 0.39 | -0.35 | -0.12 | -1.31 | -0.27 | -1.04 |
| EJ27,1 | 2.42 | 0.59 | 0.20 | -0.40 | 0.16 | -0.10 | 1.81 | 2.45 | 1.96 | 0.13 | -0.27 | -0.87 | -0.31 |
| EJ35 | 0.47 | -0.16 | -0.87 | 0.08 | -0.68 | -0.86 | 0.41 | 0.74 | 0.02 | -0.61 | -1.33 | -0.38 | -1.14 |
| Enja83 | 0.42 | -0.15 | -0.18 | -0.02 | -0.23 | 0.27 | 0.93 | 1.31 | 1.02 | 0.44 | 0.41 | 0.58 | 0.36 |
| Ee507 | 2.63 | -0.54 | -0.03 | -0.03 | -0.34 | 0.14 | 2.16 | 2.99 | 2.69 | -0.49 | 0.02 | 0.02 | -0.29 |
| Eja17 | 0.58 | -0.41 | -0.53 | 0.18 | 0.15 | -0.35 | 0.70 | 0.67 | 0.61 | -0.38 | -0.50 | 0.21 | 0.18 |
| EJ2 | 0.06 | 0.04 | -0.58 | -0.07 | -0.34 | -0.38 | -0.71 | -0.01 | -0.16 | -0.17 | -0.79 | -0.28 | -0.55 |
| Ee135 | 0.23 | 0.15 | 0.41 | 0.30 | 0.32 | -0.01 | 0.14 | 0.30 | 0.06 | -0.02 | 0.24 | 0.13 | 0.15 |
| Ee508m | 0.53 | -0.53 | -0.09 | -0.02 | 0.17 | 0.14 | 0.57 | 0.31 | 0.82 | -0.24 | 0.20 | 0.27 | 0.47 |
| Ee2-165b | 0.54 | 0.80 | 0.33 | 1.27 | 1.10 | 0.08 | -0.28 | -0.23 | 0.19 | 0.45 | -0.02 | 0.92 | 0.75 |

|  | **ANC vs** |  |  |  |  |  |  | **DUG vs** |  |  |  |  |  |
| --- | --- | --- | --- | --- | --- | --- | --- | --- | --- | --- | --- | --- | --- |
| Locus | DUG | JAB | RIJ | PEA | PEB | SPE | CDG | JAB | RIJ | PEA | PEB | SPE | CDG |
| Ee91b | 0.27 | 0.07 | 0.08 | 0.16 | -0.34 | -0.29 | 0.47 | -0.20 | -0.19 | -0.12 | -0.62 | -0.57 | 0.20 |
| Ee407 | -0.55 | 0.03 | 0.49 | -0.82 | 0.64 | -0.92 | -1.04 | 0.57 | 1.03 | -0.27 | 1.18 | -0.37 | -0.50 |
| EJ41,1 | 0.81 | 0.34 | -0.39 | 0.05 | 0.15 | 0.34 | 0.78 | -0.47 | -1.20 | -0.75 | -0.66 | -0.46 | -0.03 |
| Ee10 | 1.07 | 1.47 | 0.73 | 0.96 | -0.23 | 0.80 | 0.04 | 0.40 | -0.34 | -0.11 | -1.30 | -0.26 | -1.03 |
| EJ27,1 | 1.91 | 2.55 | 2.06 | 0.23 | -0.17 | -0.76 | -0.21 | 0.64 | 0.15 | -1.68 | -2.08 | -2.67 | -2.12 |
| EJ35 | 1.26 | 1.60 | 0.87 | 0.24 | -0.47 | 0.48 | -0.28 | 0.33 | -0.39 | -1.02 | -1.73 | -0.78 | -1.54 |
| Enja83 | 0.66 | 1.04 | 0.75 | 0.17 | 0.14 | 0.30 | 0.09 | 0.38 | 0.08 | -0.50 | -0.52 | -0.36 | -0.57 |
| Ee507 | 2.02 | 2.85 | 2.55 | -0.63 | -0.12 | -0.12 | -0.43 | 0.83 | 0.53 | -2.64 | -2.13 | -2.13 | -2.44 |
| Eja17 | 1.05 | 1.02 | 0.96 | -0.03 | -0.15 | 0.56 | 0.53 | -0.03 | -0.09 | -1.08 | -1.20 | -0.49 | -0.52 |
| EJ2 | -0.33 | 0.37 | 0.22 | 0.21 | -0.41 | 0.09 | -0.17 | 0.70 | 0.55 | 0.54 | -0.08 | 0.43 | 0.16 |
| Ee135 | 0.16 | 0.32 | 0.08 | -0.01 | 0.25 | 0.15 | 0.16 | 0.16 | -0.08 | -0.16 | 0.10 | -0.01 | 0.01 |
| Ee508m | 0.43 | 0.17 | 0.69 | -0.38 | 0.07 | 0.13 | 0.33 | -0.26 | 0.26 | -0.81 | -0.36 | -0.30 | -0.10 |
| Ee2-165b | -0.36 | -0.31 | 0.11 | 0.37 | -0.10 | 0.84 | 0.67 | 0.05 | 0.47 | 0.73 | 0.26 | 1.19 | 1.03 |

|  | **JAB vs** |  |  |  |  | **RIJ vs** |  |  |  | **PEA vs** |  |  | **PEB vs** |  | **SPE vs** |
| --- | --- | --- | --- | --- | --- | --- | --- | --- | --- | --- | --- | --- | --- | --- | --- |
| Locus | RIJ | PEA | PEB | SPE | CDG | PEA | PEB | SPE | CDG | PEB | SPE | CDG | SPE | CDG | CDG |
| Ee91b | 0.01 | 0.08 | -0.42 | -0.36 | 0.40 | 0.07 | -0.43 | -0.38 | 0.39 | -0.50 | -0.45 | 0.32 | 0.05 | 0.82 | 0.76 |
| Ee407 | 0.46 | -0.84 | 0.61 | -0.94 | -1.07 | -1.30 | 0.15 | -1.41 | -1.53 | 1.45 | -0.10 | -0.23 | -1.55 | -1.68 | -0.13 |
| EJ41,1 | -0.73 | -0.28 | -0.19 | 0.01 | 0.44 | 0.45 | 0.54 | 0.74 | 1.17 | 0.10 | 0.29 | 0.72 | 0.19 | 0.63 | 0.43 |
| Ee10 | -0.74 | -0.51 | -1.70 | -0.67 | -1.43 | 0.23 | -0.96 | 0.08 | -0.69 | -1.19 | -0.16 | -0.92 | 1.03 | 0.27 | -0.76 |
| EJ27,1 | -0.49 | -2.32 | -2.71 | -3.31 | -2.75 | -1.83 | -2.22 | -2.82 | -2.26 | -0.40 | -1.00 | -0.44 | -0.60 | -0.04 | 0.56 |
| EJ35 | -0.72 | -1.35 | -2.07 | -1.12 | -1.87 | -0.63 | -1.34 | -0.39 | -1.15 | -0.71 | 0.24 | -0.52 | 0.95 | 0.19 | -0.76 |
| Enja83 | -0.29 | -0.87 | -0.90 | -0.74 | -0.95 | -0.58 | -0.61 | -0.44 | -0.65 | -0.03 | 0.14 | -0.08 | 0.17 | -0.05 | -0.21 |
| Ee507 | -0.30 | -3.47 | -2.96 | -2.96 | -3.27 | -3.17 | -2.66 | -2.66 | -2.97 | 0.51 | 0.51 | 0.20 | 0.00 | -0.31 | -0.31 |
| Eja17 | -0.06 | -1.05 | -1.17 | -0.46 | -0.49 | -0.99 | -1.11 | -0.40 | -0.43 | -0.12 | 0.59 | 0.56 | 0.71 | 0.68 | -0.03 |
| EJ2 | -0.15 | -0.16 | -0.78 | -0.28 | -0.54 | -0.01 | -0.63 | -0.13 | -0.39 | -0.62 | -0.11 | -0.38 | 0.50 | 0.24 | -0.27 |
| Ee135 | -0.24 | -0.32 | -0.06 | -0.17 | -0.15 | -0.08 | 0.18 | 0.07 | 0.09 | 0.26 | 0.15 | 0.17 | -0.11 | -0.09 | 0.02 |
| Ee508m | 0.51 | -0.55 | -0.11 | -0.04 | 0.16 | -1.07 | -0.62 | -0.56 | -0.36 | 0.45 | 0.51 | 0.71 | 0.06 | 0.26 | 0.20 |
| Ee2-165b | 0.42 | 0.68 | 0.21 | 1.15 | 0.98 | 0.26 | -0.21 | 0.73 | 0.56 | -0.47 | 0.46 | 0.30 | 0.94 | 0.77 | -0.17 |
