# Supplementary material for: Biocomplexity in Populations of European Anchovy in the Adriatic Sea
Source: PLoS One. 2016 Apr 13;11(4):e0153061. doi: 10.1371/journal.pone.0153061 (PMC4830579; doi:10.1371/journal.pone.0153061)
Supplement: S7 Table — Samples were divided into groups according to the bottom depth. (DOCX) [file pone.0153061.s011.docx]

**S7_Table.** AMOVA on microsatellite DNA. Samples were divided into groups according to the bottom depth.

| Microsatellite DNA AMOVA | |  |  |  |  |
| --- | --- | --- | --- | --- | --- |
| Groups | Source of variation | df | Sum of squares | Variance components | % of variation |
|  |  |  |  |  |  |
| Bottom depth | among groups | 1 | 14.777 | 0.0131 | 0.35 |
|  |  |  |  |  |  |
|  | between populations within groups | 11 | 93.750 | **0.0670** | 1.79 |
|  |  |  |  |  |  |
|  | within populations | 931 | 3413.868 | **3.6668** | 97.86 |

S7_Table. df = freedom degree. Bold values are significant (P<0.05).
